# PDGFR-induced autocrine SDF-1 signaling in cancer cells promotes metastasis in advanced skin carcinoma

**DOI:** 10.1038/s41388-019-0773-y

**Published:** 2019-03-15

**Authors:** Adrià Bernat-Peguera, Pilar Simón-Extremera, Victoria da Silva-Diz, Mikel López de Munain, Laura Díaz-Gil, Rosa M. Penin, Eva González-Suárez, Diana Pérez Sidelnikova, Oriol Bermejo, Joan Maria Viñals, Francesc Viñals, Purificación Muñoz

**Affiliations:** 1 Cancer Epigenetics and Biology Program (PEBC), Bellvitge Biomedical Research Institute (IDIBELL), Barcelona, Spain; 2 Pathology Service, Hospital Universitario de Bellvitge/IDIBELL, Barcelona, Spain; 3 Plastic Surgery Unit, Hospital Universitario de Bellvitge/IDIBELL, Barcelona, Spain; 4 Laboratory of Translational Research, Catalan Institute of Oncology (ICO/IDIBELL), Barcelona, Spain; 5 Unitat de Bioquímica i Biologia Molecular, Departament de Ciències Fisiològiques, Universitat de Barcelona-IDIBELL, Barcelona, Spain; 6 Present Address: Rutgers Cancer Institute of New Jersey, Rutgers University, New Brunswick, NJ USA

**Keywords:** Squamous cell carcinoma, Cancer stem cells

## Abstract

Advanced and undifferentiated skin squamous cell carcinomas (SCCs) exhibit aggressive growth and enhanced metastasis capability, which is associated in mice with an expansion of the cancer stem-like cell (CSC) population and with changes in the regulatory mechanisms that control the proliferation and invasion of these cells. Indeed, autocrine activation of PDGFRα induces CSC invasion and promotes distant metastasis in advanced SCCs. However, the mechanisms involved in this process were unclear. Here, we show that CSCs of mouse advanced SCCs (L-CSCs) express CXCR4 and CXCR7, both receptors of SDF-1. PDGFRα signaling induces SDF-1 expression and secretion, and the autocrine activation of this pathway in L-CSCs. Autocrine SDF-1/CXCR4 signaling induces L-CSC proliferation and survival, and mediates PDGFRα-induced invasion, promoting in vivo lung metastasis. Validation of these findings in patient samples of skin SCCs shows a strong correlation between the expression of *SDF1*, *PDGFRA*, and *PDGFRB*, which is upregulated, along CXCR4 in tumor cells of advanced SCCs. Furthermore, PDGFR regulates SDF-1 expression and inhibition of SDF-1/CXCR4 and PDGFR pathways blocks distant metastasis of human PD/S-SCCs. Our results indicate that functional crosstalk between PDGFR/SDF-1 signaling regulates tumor cell invasion and metastasis in human and mouse advanced SCCs, and suggest that CXCR4 and/or PDGFR inhibitors could be used to block metastasis of these aggressive tumors.

## Introduction

Squamous cell carcinoma (SCC) is the second most common non-melanoma skin cancer in humans, accounting for 20% of cutaneous malignancies [1, 2]. Most invasive skin SCCs conserve epithelial differentiation traits and are considered to be well-differentiated SCCs (WD-SCCs). A subset of SCCs show poorly differentiated features and eventually become spindle-shaped (PD/S-SCCs), the latter characteristic being associated with enhanced recurrence, metastasis, and reduced patient survival [3–5]. Most skin SCC lesions are treated by surgical excision. Although high risk and metastatic skin SCCs are treated with adjuvant radiotherapy or chemotherapy, the clinical benefits of these treatments have been limited [6]. Therefore, it is important to design targeted therapies that efficiently block the aggressive growth and metastasis of advanced SCCs.

Mouse skin SCC cells expressing CD34 and α6-integrin, or Sox2 are enriched in tumor-initiating or cancer stem-like cells (CSCs) relative to the bulk of tumor cells. This cell population is responsible for long-term SCC growth and metastasis [7–9]. Highly aggressive and metastatic mouse PD/S-SCCs arise from the malignant progression of WD-SCCs [10, 11] and during this process, the CSC population is dramatically expanded and regulatory mechanisms controlling CSC proliferation and dissemination change. FGFR1 signaling is induced in the CSCs of advanced PD/S-SCCs to promote aggressive growth. The PDGFRα pathway is also activated by an autocrine way in these CSCs, which promotes CSC invasion and enhances metastasis in PD/S-SCCs [11]. However, the mechanisms involved in PDGFRα-induced metastasis remain unclear.

The chemokine SDF-1 (CXCL12), which binds to G protein-coupled receptors CXCR4 and CXCR7, plays an important role in tumor growth and metastasis in different tumor types [12–15]. Stromal fibroblasts and cancer cells produce SDF-1 [16], which stimulates cancer cell proliferation and is responsible for recruiting CXCR4-expressing endothelial progenitor cells, thereby increasing tumor angiogenesis [17, 18]. CXCR4 is expressed by CSCs in various tumor types [19–21]. SDF-1/CXCR4 signaling increases the self-renewal of breast and brain CSCs [22–24], and the epithelial-to-mesenchymal transition (EMT) program and metastasis in sarcomas, breast, pancreatic, colon, and liver cancer cells [24–27]. CXCR7/RDC1 receptor is expressed by immune, endothelial, and tumor cells and binds SDF-1 with high affinity [28]. Although CXCR7 was thought to act as a scavenger receptor [29], it was recently demonstrated that SDF-1/CXCR7 signaling induces CSC proliferation/survival and EMT [30, 31], supporting growth and metastasis in different tumor types.

SDF-1 is significantly upregulated in stromal fibroblasts of human skin SCCs relative to normal skin [32], and CXCR4 is upregulated in metastatic SCCs in comparison to normal skin and non-metastatic skin carcinomas [33], whereas CXCR7 expression is induced in 70% of patient skin SCC samples and is associated with cancer cell survival [34]. However, the role of the SDF1/CXCR4/CXCR7 pathway in cutaneous SCCs has been unclear. Here, we demonstrate that functional crosstalk between PDGFR/SDF-1 pathways induces the autocrine activation of SDF-1/CXCR4 signaling in cancer cells of mouse and human PD/S-SCCs, which promotes distant metastasis.

## Results

### SDF-1 and CXCR4 expression is upregulated in mouse advanced SCCs

**Fig. 1:**
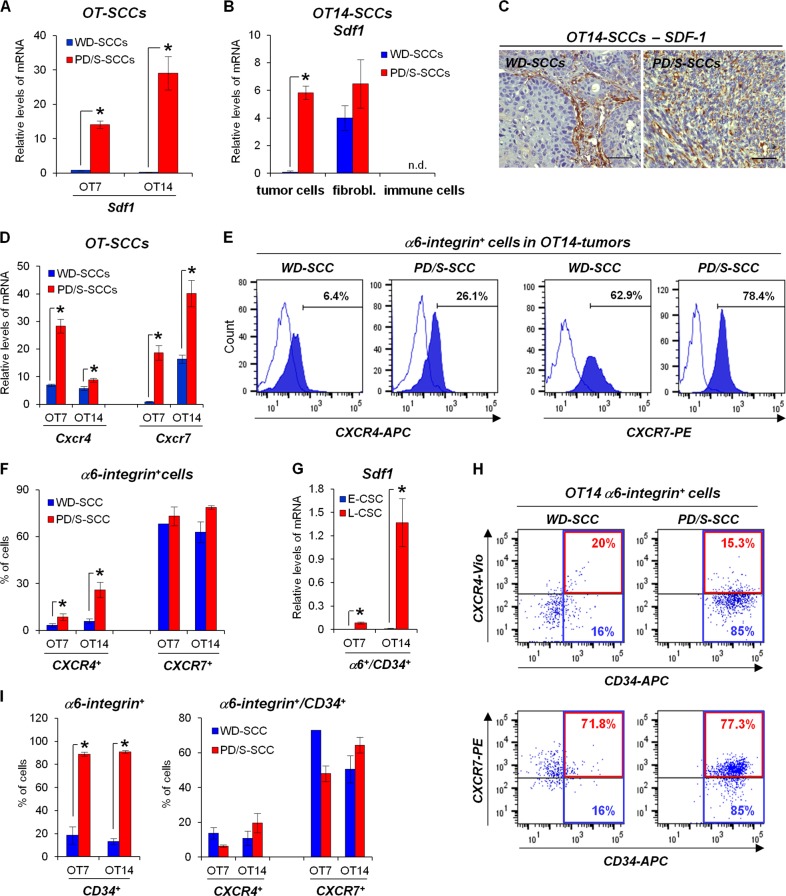
CSCs of mouse advanced skin SCCs express CXCR4 and CXCR7 and up-regulate the expression of *Sdf1*. **a**, **b** Mean (±SE) levels of *Sdf1* mRNA relative to *Gapdh*, as quantified by qRT-PCR in WD-SCCs and PD/S-SCCs (three different samples per group) of OT7 and OT14 lineages (**a**), and in tumor cells (α6-integrin^+^/CD45^−^/CD31^−^ cells), immune cells (α6-integrin^−^/EpCAM^−^/CD45^+^/CD31^−^ cells), and fibroblasts (α6-integrin^−^/EpCAM^−^/CD45^−^/CD31^−^ cells) isolated from OT14 WD-SCCs and PD/S-SCCs (two different samples per group) by FACS-sorter (**b**). **c** Representative images of the SDF-1 immunodetection in paraffin sections of WD-SCCs and PD/S-SCCs of OT14 lineage. Scale bar, 100 μm. **d** Mean (±SE) levels of the indicated mRNAs, relative to *Gapdh*, as quantified by qRT-PCR in WD-SCCs and PD/S-SCCs (three different samples per group) of OT7 and OT14 lineages. **e** Representative results of quantification of the indicated cell populations in OT14 WD-SCCs and PD/S-SCCs by flow cytometry. Percentage of α6-integrin^+^/CXCR4^+^ and α6-integrin^+^/CXCR7^+^ cells is indicated in each panel. **f** Mean percentages (±SE) of cell populations analyzed in **e** (3–10 different tumor samples per group). **g** Mean (±SE) *Sdf1* mRNA levels relative to *Gapdh* in E-CSCs and L-CSCs (three different samples per group) isolated by FACS-sorter from the indicated tumors. **h** Flow cytometry quantification of CXCR4^+^ and CXCR7^+^ cells (red numbers) into the α6-integrin^+^/CD34^+^ CSC population (blue numbers) of the indicated tumors. **i** Mean percentage (±SE) of the indicated cell populations in WD-SCCs and PD/S-SCCs, as quantified in **h**. *, significant differences between compared groups (*t*-test; *P* ≤ 0.05). n.d. not detected

To determine the relevance of SDF-1 signaling in skin SCC growth and metastasis, we initially compared the expression of this chemokine and *Cxcr4* and *Cxcr7* receptors in early WD-SCCs and advanced PD/S-SCCs of two different lineages of mouse skin SCC progression (OT7 and OT14). In each of these lineages, PD/S-SCCs were generated after the serial engraftment of their WD-SCC precursors in immunodeficient mice [11]. We found that *Sdf1* expression, which was weakly detected in early SCCs, was upregulated in PD/S-SCCs (Fig. 1a). In WD-SCCs, *Sdf1* was expressed by fibroblasts (Fig. 1b) and immunodetected in stromal cells (Fig. 1c), as previously described [32, 35], whereas tumor cells and CD45^+^ immune cells (deficient in T-cell in nude immunodeficient mice) exhibited a faint or undetectable *Sdf1* expression (Fig. 1b). In contrast, SDF-1 was strongly upregulated in tumor cells of PD/S-SCCs (Fig. 1c), reaching similar levels to those expressed by fibroblasts (Fig. 1b).

*Cxcr4* and *Cxcr7* expression was upregulated in advanced SCCs relative to WD-SCCs (Fig. 1d). An expansion of CXCR4-expressing tumor cells was detected by immunohistochemistry in PD/S-SCCs, as compared to WD-SCCs (Supplementary Fig. 1A). Flow cytometry analysis showed that around 3–7% of tumor cells expressed CXCR4 (α6-integrin^+^/CXCR4^+^ cells) in WD-SCCs, and this frequency was significantly increased in PD/S-SCCs (Fig. 1e, f). CXCR7^+^ cells were more frequent than CXCR4-expressing cells in early and advanced SCCs (Supplementary Fig. 1A and 1B). Accordingly, 60–70% of tumor cells expressed CXCR7 (α6-integrin^+^/CXCR7^+^ cells) in WD-SCCs and this frequency was not significantly increased in PD/S-SCCs (Fig. 1e, f). These results suggest that increased levels of *Cxcr7* mRNA detected in advanced tumors may be associated with a different stromal/tumor cells ratio in WD-SCCs and PD/S-SCCs [36, 37].

Analysis of the ligand and receptors in the CSC population (α6-integrin^+^/CD34^+^ cells) showed that CSCs isolated from PD/S-SCCs (L-CSCs) strongly expressed *Sdf1*, whereas the expression of this chemokine was practically undetectable in CSCs of WD-SCCs (E-CSCs) (Fig. 1g). CXCR4 was mostly expressed by a subpopulation (5–20%) of E-CSCs (Fig. 1h, i), whereas CXCR7 was detected in CSC and non-CSC populations, and around 50–80% of E-CSCs expressed CXCR7. Furthermore, the percentage of CXCR4- or CXCR7-expressing α6-integrin^+^/CD34^+^ cells was not significantly altered in PD/S-SCCs compared with WD-SCCs (Fig. 1h, i). Since the α6-integrin^+^/CD34^+^-CSC population is expanded in advanced tumors (Fig. 1i; see [7, 11]), our results indicate that the global amount of CXCR4^+^-CSCs was increased in the PD/S-SCCs.

It is important to highlight that similar *Sdf1* and *Cxcr4* upregulation was observed in primary PD/S-SCCs (spontaneously developed in K14-HPV16 mice [11]) and in PD/S-SCCs that were engrafted in syngeneic immunocompetent mice, relative to their respective WD-SCCs (Supplementary Fig. 1C and 1D). SDF-1 expression was significantly induced in tumor and stromal cells (Supplementary Fig. 1E), and an expansion of CXCR4-expressing CSCs was detected in PD/S-SCCs generated in immunocompetent mice (Supplementary Fig. 1F–1H), indicating that SDF-1 signaling may be activated in CSCs of advanced tumors independently of the immune status of the mice.

### Autocrine SDF-1 signaling promotes PD/S-SCC CSC proliferation and invasion

**Fig. 2:**
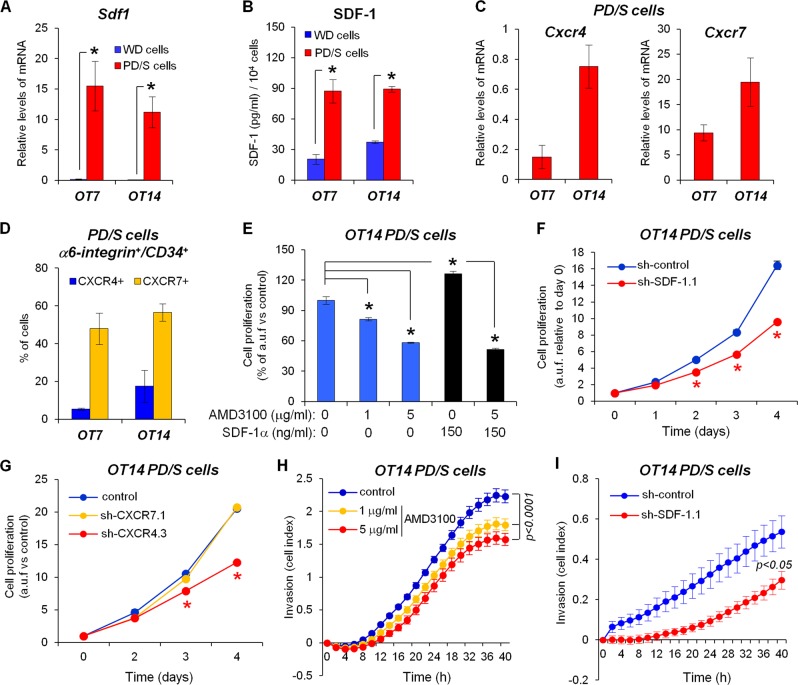
Autocrine SDF-1 signaling promotes L-CSC proliferation and invasion. **a** Mean (±SE) of *Sdf1* mRNA levels relative to *Gapdh*, as quantified by qRT-PCR in in vitro growing tumor cells isolated from WD-SCC (WD cells) and PD/S-SCCs (PD/S cells) of the indicated tumor lineages (two different primary cell cultures per tumor type and lineage). **b** SDF-1 concentration in the culture medium of the indicated cells (two primary culture cells per group and lineage), as quantified by ELISA assays. **c** Mean (±SE) *Cxcr4* and *Cxcr7* mRNA levels, relative to *Gapdh*, in PD/S cell cultures (three samples per group) of the indicated lineages. **d** Mean percentages (±SE) of CXCR4- or CXCR7-expressing α6-integrin^+^/CD34^+^ cells (three samples per group) in PD/S cells of OT7 and OT14 lineages, as quantified by flow cytometry. **e** Cell proliferation upon SDF-1α and/or AMD3100 treatment, as measured by MTT. Results show means (±SE) of arbitrary units of fluorescence (a.u.f.) in treated cells relative to cells growing without SDF-1α and inhibitor. **f** Proliferation kinetics (means±SE of a.u.f. relative to day 0) of control PD/S cells (sh-control) or SDF-1 interfered cells (sh-SDF-1.1), as measured by MTT. Representative results of two independent assays. **g** Proliferation kinetics (means±SE of a.u.f.) of control PD/S cells (sh-control) and PD/S cells with CXCR4 (sh-CXCR4.3) or CXCR7 (sh-CXCR7.1) mRNA interference, as measured by MTT. Representative results of three independent assays. **h**, **i** Comparison of invasion capacity (mean cell index±SE) of PD/S cells upon control or AMD3100 treatment (**h**), or in SDF-1 interfered PD/S cells (**i**) in x-CELLingence real-time analysis. Representative results of two independent assays. *, significant differences between the compared groups (*t*-test; *P* ≤ 0.05)

To determine whether an autocrine SDF-1 signaling is induced in L-CSCs, we isolated tumor cells from WD-SCCs (WD cells) and PD/S-SCCs (PD/S cells), which were maintained in culture. We previously demonstrated that these primary cultures were enriched in tumor-initiating cells that conserved the molecular traits of parental SCC CSCs [11]. In this regard, PD/S cells expressed and secreted higher levels of SDF-1 than WD cells (Fig. 2a, b). In addition, PD/S cells expressed *Cxcr4* and *Cxcr7*, showing a lower expression of *Cxcr4* than *Cxcr7* (Fig. 2c). Accordingly, 40–60% of α6-integrin^+^/CD34^+^-CSCs in PD/S cell cultures expressed CXCR7, whereas 6–16% of α6-integrin^+^/CD34^+^-CSCs expressed CXCR4 (Fig. 2d; Supplementary Fig. 2A and 2B), and most CXCR4^+^-CSCs expressed CXCR7 (Supplementary Fig. 2C).

To determine the impact of SDF-1 signaling on proliferation, PD/S cells growing in basal medium or supplemented with purified chemokine were treated with AMD3100, an antagonist of CXCR4 [38]. AMD3100 treatment significantly inhibited the proliferation/survival of PD/S cells under basal conditions, and addition of SDF-1 to the medium did not alter or only slightly induced the proliferation of PD/S cells (Fig. 2e; Supplementary Fig. 2D). Similarly, the proliferation of PD/S cells with interfered expression of SDF-1 (Supplementary Fig. 2E) was significantly reduced in comparison with that of PD/S control cells (Fig. 2f; Supplementary Fig. 2F), indicating that the autocrine activation of SDF-1 signaling promotes PD/S cell proliferation. The expression of *Cxcr4*, but not of *Cxcr7*, was significantly reduced in SDF-1 knocked-down cells (Supplementary Fig. 2G), suggesting that *Cxcr4* expression is regulated directly or indirectly by SDF-1 signaling.

PD/S cells showed in vitro a strong migration and invasion capacity, which was associated with enhanced distant metastasis in advanced SCCs [11]. We observed that AMD3100 treatment as well as SDF-1 abrogation significantly reduced the invasive capability of PD/S cells (Fig. 2h, i). These results indicate that autocrine SDF-1 signaling promotes CSC motility and invasion, which may consequently favor CSC dissemination and metastasis.

### CXCR4 inhibition blocks distant metastasis in mouse PD/S-SCCs

As SDF-1 signaling can be activated through CXCR4 and CXCR7, we evaluated the role of each of these receptors in regulating PD/S-SCC growth and metastasis. For this purpose, we knocked down the expression of *Cxcr4* and *Cxcr7* in PD/S cells of different SCC lineages (Supplementary Fig. 3A). Abrogation of CXCR4 did not affect the expression of *Cxcr7*, but did significantly reduce the expression of *Sdf1* (Supplementary Fig. 3B). In contrast, CXCR7 knockdown induced the downregulation of *Cxcr4*, whereas *Sdf1* expression was significantly reduced only in OT7 PD/S cells (Supplementary Fig. 3B). As OT7 cells showed a lower level of *Cxcr4* expression and a smaller percentage of CXCR4^+^-CSCs than OT14 cells (Fig. 2c, d), CXCR7 knockdown may lead to an almost complete abrogation of *Cxcr4* in OT7 cells, in turn causing a significant downregulation of *Sdf1*. Therefore, these data suggest that the expression of *Cxcr4* is finely regulated by the levels of SDF-1 and CXCR7 and, in turn, that CXCR4 regulates the expression of the ligand.

CXCR4 knockdown significantly reduced the proliferation of PD/S cells in both lineages, whereas CXCR7 knockdown only inhibited the proliferation of OT7 PD/S cells, in accordance with the strong downregulation of *Sdf1* observed in these cells (Fig. 2g; Supplementary Fig. 3C).

**Fig. 3:**
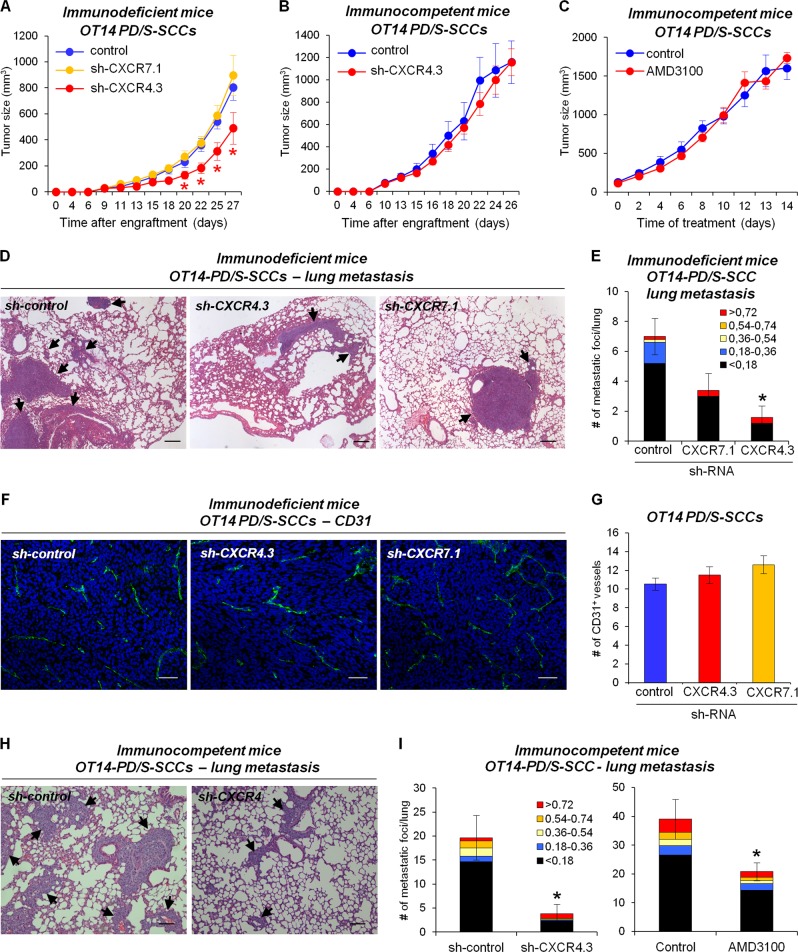
CXCR4 inhibition in L-CSCs blocks mouse PD/S-SCC metastasis. **a**, **b** Growth kinetics (means±SE of tumor size, mm^3^) of control (sh-control), CXCR4 (sh-CXCR4.3), and CXCR7 knocked-down (sh-CXCR7.1) PD/S-SCCs (eleven tumors per group) in immunodeficient mice (**a**), and of sh-control and sh-CXCR4.3 PD/S-SCCs (six and ten tumors, respectively) in syngeneic immunocompetent mice (**b**). **c** Growth kinetics (means ± SE of tumor size, mm^3^) of control and AMD3100 treated PD/S-SCCs (seven tumors per group) growing in immunocompetent mice. **d** Metastatic lesions (indicated by black arrows) in the lungs of immunodeficient mice carrying sh-control, sh-CXCR4.3, and sh-CXCR7.1 PD/S-SCCs. Scale bar, 200 μm. **e** Mean of metastatic foci (±SE) per lung section (categorized by size, mm^2^) in mice with the indicated PD/S-SCC types (5–6 mice per group), as determined in **d**. **f** Representative images of the immunodetection of CD31^+^ vessels in sh-control, sh-CXCR4.3, and sh-CXCR7.1 PD/S-SCCs. Scale bar, 40 μm. **g** Mean frequency (±SE; 3–4 tumor samples per group) of CD31^+^ vessels per tumor section, as determined in **f**. **h** Metastatic lesions (indicated by black arrows) in the lungs of immunocompetent mice carrying the sh-control and sh-CXCR4.3 PD/S-SCCs. Scale bar, 200 μm. **i** Mean of metastatic foci (±SE) per lung section (categorized by size, mm^2^) in mice with the indicated PD/S-SCC types and treatments (six mice per group). *, significant differences between the compared groups (*t*-test; *P* ≤ 0.05)

In order to determine the impact of CXCR4 and CXCR7 on PD/S-SCC growth and metastasis, we engrafted sh-control, sh-CXCR4, and sh-CXCR7 PD/S cells from the OT14 lineage in immunodeficient mice. sh-CXCR4 tumors showed a reduced percentage of α6-integrin^+^/CXCR4^+^ tumor cells, and specifically of CXCR4-expressing CSCs, compared with sh-control tumors, whereas the percentage of CXCR7-expressing CSCs was not affected (Supplementary Fig. 3D–3F). In contrast to CXCR7 knocked-down tumors, sh-CXCR4 tumors grew less than control tumors (Fig. 3a), in accordance with the significant reduction in the percentage of proliferating tumor cells observed in the CXCR4-deficient tumors (Supplementary Fig. 3G and 3H). To determine the impact of CXCR4 signaling on tumor growth under an immunocompetent background, sh-control and sh-CXCR4 PD/S cells were engrafted in syngeneic immunocompetent mice. In contrast to that observed in immunodeficient mice, tumor growth was not significantly blocked after CXCR4 abrogation (Fig. 3b; Supplementary Fig. 3I), or after AMD3100 treatment (Fig. 3c), suggesting that the effect of CXCR4 inhibition on tumor growth may be tumor microenvironment-dependent.

Furthermore, immunodeficient mice carrying sh-CXCR4 PD/S-SCCs developed significantly less metastatic foci in the lungs than mice carrying sh-control tumors (Fig. 3d, e), in the absence of alterations in the tumor angiogenesis, as indicated by the unaltered frequency of CD31^+^ vessels (Fig. 3f, g) and the conserved expression of *Vegfr2*, a marker of endothelial cells (Supplementary Fig. 3J). Despite the number of metastases generated from CXCR7-interfered PD/S-SCCs was lower than from sh-control tumors, this difference was not significant (Fig. 3d, e) and could be associated with the downregulation of *Cxcr4* expression induced by CXCR7 knockdown.

Similarly, CXCR4 abrogation in L-CSCs and CXCR4 pharmacological inhibition by AMD3100 treatment significantly reduced lung metastasis development in immunocompetent mice (Fig. 3h, i). Taken together, these results indicate that CXCR4 inhibition blocks distant metastasis of PD/S-SCCs, independently of the immune status of mice.

### SDF-1 abrogation in L-CSCs inhibits distant metastasis

**Fig. 4:**
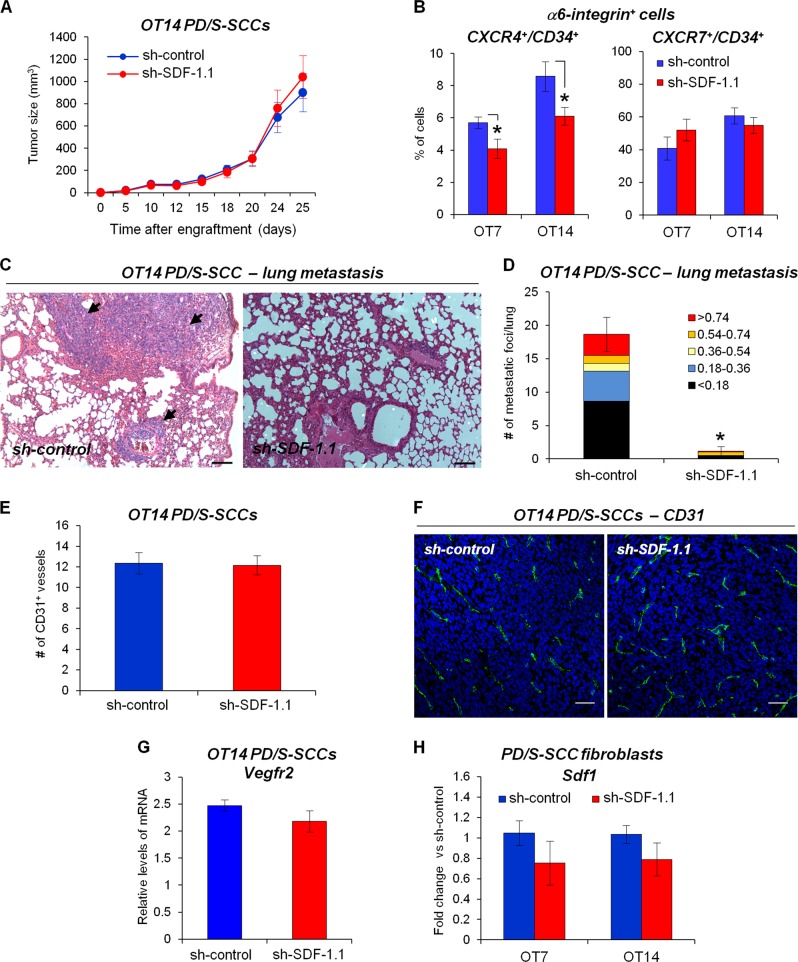
Inhibition of autocrine SDF-1 signaling in L-CSCs blocks metastasis development. **a** Growth kinetics (mean ± SE of tumor size, mm^3^) of SDF-1 expressing (sh-control) or SDF-1 knocked-down (sh-SDF-1.1) PD/S-SCCs (eleven tumors per group). **b** Mean percentage (±SE) of CXCR4- or CXCR7-expressing α6-integrin^+^/CD34^+^ CSCs in the indicated tumor (7–9 samples per tumor group). **c** Metastatic lesions (indicated by black arrow) in lungs of immunodeficient mice carrying sh-control or sh-SDF-1.1 PD/S-SCCs. Scale bar, 200 μm. **d** Mean of metastatic foci (±SE) per lung section (categorized by size, mm^2^) in mice with the indicated PD/S-SCC types (six mice per group), as determined in **c**. **e** Mean frequency (±SE; 4–6 samples per tumor group) of CD31^+^ vessels per tumor section in sh-control and sh-SDF-1.1 PD/S-SCCs. **f** Representative images of the immunodetection of CD31^+^ vessels in the indicated tumors. Scale bar, 40 μm. **g** Mean level (±SE) of *Vegfr2* mRNA, relative to *Gapdh* in the indicated tumors (4–6 samples per tumor group). **h** Mean level (±SE) of *Sdf1* mRNA in fibroblasts of sh-SDF-1 PD/S-SCCs, relative to that detected in fibroblasts of sh-control tumors. *, significant differences between the compared groups (*t*-test; *P* ≤ 0.05)

To determine the impact of *Sdf1* upregulation in L-CSCs on PD/S-SCC growth and metastasis, we engrafted sh-control and sh-SDF-1 PD/S cells in immunodeficient mice. Tumor latency and growth kinetics were similar in sh-control and sh-SDF-1 PD/S-SCCs (Fig. 4a; Supplementary Fig. 4A), even though the interference of the chemokine expression in tumor cells was maintained during tumor growth (Supplementary Fig. 4B). Furthermore, a reduction in the percentage of α6-integrin^+^/CD34^+^/CXCR4^+^ CSCs, but not of α6-integrin^+^/CD34^+^/CXCR7^+^ CSCs, was observed in sh-SDF-1 tumors, compared with sh-control tumors (Fig. 4b; Supplementary Fig. 4C and 4D), in accordance with the downregulated expression of *Cxcr4* observed in sh-SDF-1-interfered PD/S cells (Supplementary Fig. 2G).

SDF-1 abrogation dramatically reduced the development of lung metastasis from PD/S-SCCs (Fig. 4c, d; Supplementary Fig. 4E). As stroma-derived SDF-1 promotes the recruitment of endothelial cells at tumors [39], SDF-1 interference in tumor cells might also alter tumor angiogenesis, blocking metastasis. However, a similar density of CD31^+^ vessels and *Vegfr2* expression were observed in control and SDF-1-interfered tumors (Fig. 4e–g). Metastasis blockage was not related to the attenuation of the EMT program, as the expression of EMT-inducer transcription factors (Supplementary Fig. 4F) and the repressed expression of *Cdh1* (E-Cadherin) (data not shown) was not altered in sh-SDF-1 tumor cells. No changes in the expression of SDF-1 were detected in the fibroblasts isolated from sh-SDF-1 PD/S-SCCs, as compared with the control tumors (Fig. 4h). These results suggest that a paracrine activation of SDF-1 signaling or the activation of other stroma-induced signaling pathways can support tumor cell proliferation, but not distant metastasis.

Taken together, these results indicate that abrogation of autocrine SDF-1 signaling reduces the migration and invasion capacity of CSCs of PD/S-SCCs, blocking the development of metastasis of these advanced tumors.

### Functional PDGFRα/SDF-1 signaling crosstalk promotes L-CSC invasion in mouse PD/S-SCCs

**Fig. 5:**
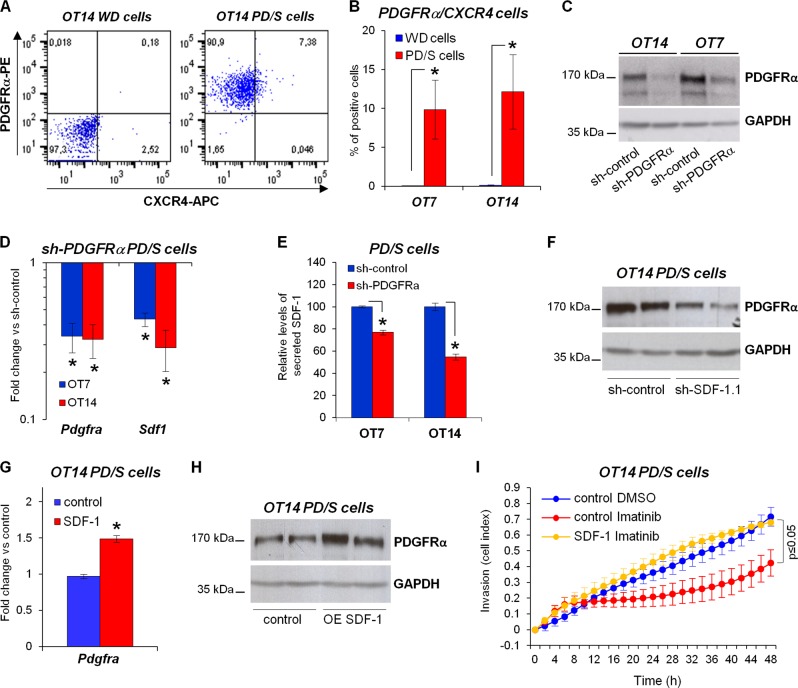
PDGFRα-induced invasion in L-CSCs is mediated by SDF-1 signaling. **a** Representative results of the quantification by flow cytometry of the percentage of PDGFRα^+^/CXCR4^+^ cells in WD and PD/S cells. Percentage of each cell population is indicated. **b** Mean percentage (±SE; 3–5 different cell cultures per group and lineage) of PDGFRα^+^/CXCR4^+^ cells in the indicated cell cultures, as analyzed in **a**. **c** Representative images of PDGFRα expression in the indicated cells after transduction with sh-control and sh-PDGFRα lentivirus. **d** Mean mRNA levels (±SE) of the indicated genes in sh-PDGFRα PD/S cells relative to sh-control PD/S cells of OT7 and OT14 tumor lineages (three different samples per group). **e** Quantification of SDF-1 concentration (mean±SE) in the culture medium of the indicated PD/S cells by ELISA assays (three samples per group). **f** Representative images of PDGFRα expression in two independent samples of control and SDF-1-interfered PD/S cells. **g** Mean *Pdgfra* mRNA levels (±SE) in SDF-1-overexpressing PD/S cells (SDF-1) relative to control cells (transduced with empty vector). **h** Representative images of PDGFRα expression in two independent samples of control and SDF-1-overexpressing PD/S cells (OE SDF-1). **i** Representative results of the invasion capability (mean cell index ± SE) of control PD/S cells in the absence (control DMSO) or presence of Imatinib (4 μM) (control Imatinib) and in Imatinib-treated SDF-1-overexpressing PD/S (SDF-1 Imatinib), as measured by x-CELLingence real-time analysis. GAPDH was used as a protein-loading control in **c**, **f** and **h**. *, significant differences between the compared groups (*t*-test; *P* ≤ 0.05)

PDGFRα signaling promotes L-CSC invasion and PD/S-SCC metastasis [11], so we tested whether activation of this pathway regulates SDF-1 signaling in SCC CSCs. Flow cytometry assays demonstrated that whereas the scarce population of CXCR4^+^ WD cells did not express PDGFRα, most PD/S cells, including CXCR4^+^ PD/S cells, exhibited high levels of PDGFRα (Fig. 5a, b). PDGFRα knockdown (Fig. 5c) significantly reduced *Sdf1* expression in PD/S cells, as determined by quantitative reverse transcriptase PCR (RT-PCR) and enzyme-linked immunosorbent assay (ELISA) assays (Fig. 5d, e). In turn, SDF-1 knockdown reduced the expression of PDGFRα (Fig. 5f; Supplementary Fig. 5A and 5B), whereas the ectopic expression of SDF-1 in PD/S cells (Supplementary Fig. 5C) further upregulated the expression of PDGFRα (Fig. 5g, h), indicating that the activity of each of these signaling pathways modulates the activity of the other. In order to determine whether PDGFRα-induced invasion of PD/S cells is mediated by SDF-1 signaling, we compared the invasion capability of these cells under control conditions, upon inhibition of PDGFRα signaling by treatment with Imatinib, an inhibitor of PDGFR, and in Imatinib-treated SDF-1α-overexpressing PD/S cells. Our results demonstrated that whereas Imatinib treatment significantly reduced the invasion of PD/S cells, the ectopic expression of SDF-1 restored the invasion of Imatinib-treated PD/S cells (Fig. 5i), indicating that PDGFRα-induced invasion is mediated by SDF-1 signaling in PD/S cells. Therefore, these results demonstrated that PDGFRα and SDF-1 signaling crosstalk regulates the invasion capability of L-CSCs and promotes metastasis development in advanced PD/S-SCCs.

### PDGFR/SDF-1 signaling crosstalk in cancer cells promotes metastasis in human advanced SCCs

**Fig. 6:**
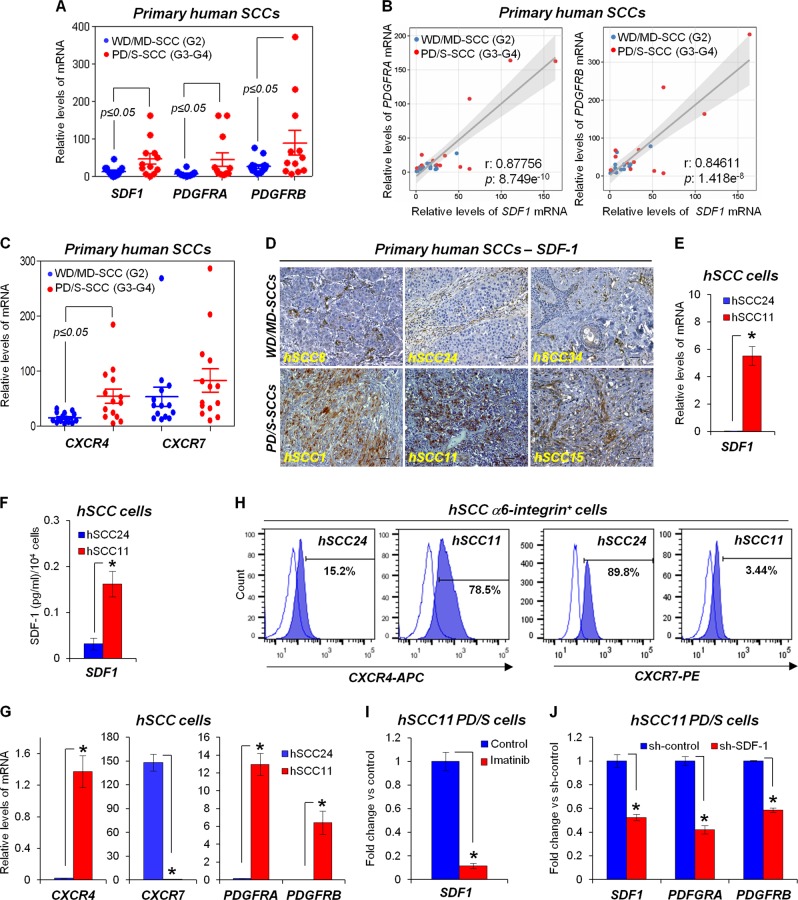
Functional crosstalk between PDGFR and SDF1/CXCR4 signaling in cancer cells of human advanced SCCs. **a** mRNA levels of the indicated genes relative to *GAPDH* (individual data and mean ± SE) in WD/MD-SCC and PD/S-SCC patient samples. *P* value (*t*-test) of the compared groups is indicated. **b** Correlation between *SDF1* and *PDGFRA* or *PDGFRB* mRNA levels in the indicated patient samples, as determined by Pearson’s test. *P* value is indicated. **c** mRNA levels of *CXCR4* and *CXCR7* (individual data and mean ± SE) in the indicated patient samples. *P* value (*t*-test) of the compared groups is indicated. **d** Representative images of the immunodetection of SDF-1 in paraffin sections of WD/MD-SCCs and PD/S-SCCs patient samples. Scale bar, 100 μm. **e** Mean (±SE) *SDF1* mRNA levels relative to *GAPDH*, in primary culture cells isolated from human WD/MD-SCC (hSCC24) and PD/S-SCC (hSCC11). **f** Mean (±SE) of SDF-1 concentration in the culture medium of the indicated cells, as determined by ELISA assays. **g** Mean (±SE) mRNA levels of the indicated genes, relative to *GAPDH*, in primary culture of hSCC24 and hSCC11 cells. **h** Quantification of the indicated cell populations in hSCC24 and hSCC11 cells by flow cytometry. Percentage of α6-integrin^+^/CXCR4^+^ and α6-integrin^+^/CXCR7^+^ cells is indicated in each panel. **i**, **j** Mean (±SE) of the indicated mRNA in Imatinib-treated (4 μM for 48 h) relative to those detected in untreated (control) hSCC11 PD/S cells (**i**), or in sh-SDF-1 relative to sh-control hSCC11 PD/S cells (**j**). *, significant differences between the compared groups (*t*-test; *P* ≤ 0.05)

Advanced and recurrent human PD/S-SCCs of skin induce the expression of *PDGFRA/B*, similarly to mouse PD/S-SCCs [11]. To determine whether *SDF1* expression is upregulated in human PD/S-SCCs, we analyzed the expression of this chemokine in a subset of patient WD/MD-SCCs (G2 grade tumors) and advanced SCCs (G3-G4 grade tumors) (Supplementary Table 1). We found that *SDF1* and *CXCR4* expression was induced in human skin SCCs at late stages of progression, whereas the expression of CXCR7 was not significantly affected (Fig. 6a, c; Supplementary Table 1). Furthermore, a high positive correlation was observed between *SDF1* and *PDGFRA* or *PDGFRB* expression in this subset of patient tumors (Fig. 6b). SDF-1 was mostly expressed by stromal cells of patient WD/MD-SCCs and was strongly induced in tumor cells of advanced SCCs (Fig. 6d), in accordance with that observed in mouse SCCs (Fig. 1c). A reduced population of tumor cells expressed CXCR4 in early tumors and this population was expanded in the patient PD/S-SCCs (Supplementary Fig. 6A).

To determine the relevance of SDF-1 signaling in human SCCs, we established primary cultures of tumor cells from a patient WD/MD-SCC (hSCC24 cells) and from a patient PD/S-SCC (hSCC11 cells). hSCC24 WD cells grew as adherent cells and exhibited a typical epithelial shape, whereas hSCC11 PD/S cells grew forming spheres (Supplementary Fig. 6B). In accordance with the conserved epithelial differentiation traits of patient WD/MD-SCCs [11], hSCC24 WD cells expressed high levels of EpCAM and E-cadherin epithelial markers, whereas hSCC11 PD/S cells lost the expression of these epithelial markers and upregulated the expression of EMT-inducer transcription factors (Supplementary Fig. 6C and 6D). These observations indicate that the EMT program was strongly induced in these human PD/S cells. hSCC11 PD/S cells induced the expression and secretion of SDF-1 (Fig. 6e, f), as well as the *CXCR4* expression, as compared to hSCC24 WD cells (Fig. 6g). Furthermore, we observed an expansion of the α6-integrin^+^/CXCR4^+^ cell population in hSCC11 PD/S cells (Fig. 6h), consistent with that observed by immunohistochemistry in patient samples. However, *CXCR7* expression was significantly downregulated in hSCC11 PD/S cells (Fig. 6g), in contrast to what was observed in mouse PD/S cells and in other human cutaneous SCC cell lines [34]. In this regard, whereas 89% of hSCC24 WD cells expressed CXCR7, this population of cells was strongly reduced in hSCC11 PD/S cells (Fig. 6h). These findings suggest that CXCR7 expression in tumor cells may be dependent on intrinsic features and/or the stage of progression of patient tumors. Therefore, these results indicate that SDF-1/CXCR4 signaling is induced in tumor cells of advanced human SCCs.

In addition, a strong upregulation of PDGFRα and PDGFRβ was detected in hSCC11 PD/S cells (Fig. 6g; Supplementary Fig. 6E), indicating that tumor cells from human advanced SCCs induce PDGFR signaling. Inhibition of PDGFR by Imatinib treatment significantly reduced the expression of *SDF1* and in turn, SDF-1 knockdown reduced *PDGFRA* and *PDGFRB* expression in hSCC11 PD/S cells (Fig. 6i, j). Therefore, PDGFR signaling controls SDF-1 expression and vice versa, establishing a functional crosstalk between PDGFR/SDF-1 signaling in tumor cells of human advanced SCCs.

**Fig. 7:**
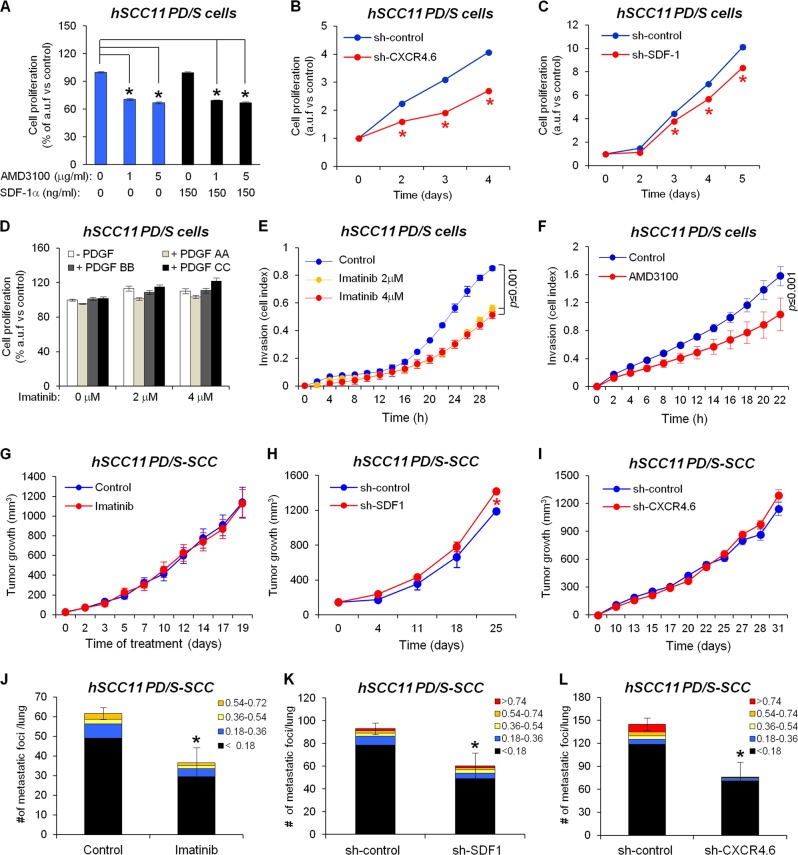
Inhibition of PDGFR and SDF1/CXCR4 signaling pathways blocks distant metastasis in human PD/S-SCCs. **a**, **d** hSCC11 cell proliferation after the indicated treatments, as measured by MTT. Mean (±SE) of arbitrary units of fluorescence (a.u.f.) of treated cells relative to cells growing without SDF-1 or AMD3100 (**a**), or without PDGF or Imatinib (**d**). **b**, **c** Representative proliferation kinetics (means±SE of a.u.f. relative to day 0) of control (sh-control) and CXCR4 knocked-down (sh-CXCR4.6) hSCC11 cells (**b**), or of control and SDF-1 interfered hSCC11 cells (sh-SDF-1) (**c**), as measured by MTT. **e**, **f** Comparison of invasion capacity (mean cell index ± SE) of hSCC11 PD/S cells upon control or Imatinib treatment (**e**), or after AMD3100 treatment (5 μg/mL) (**f**) in x-CELLingence real-time analysis. **g**–**i** Growth kinetics (mean ± SE of tumor size, mm^3^) of tumors generated after engrafting in immunodeficient mice: **g** hSCC11 cells, which were treated daily with vehicle (control) or Imatinib (nine tumors per group); **h** sh-control and SDF-1 knocked down (sh-SDF-1) hSCC11 cells (five tumors per group); and **i** sh-control and CXCR4 knocked-down (sh-CXCR4.6) hSCC11 cells (ten tumors per group). **j**–**l** Mean of metastatic foci (±SE) per lung section (categorized by size, mm^2^) developed in: **j** control and Imatinib-treated mice (six mice per group); **k** sh-control and sh-SDF-1 PD/S-SCCs carrying mice (five mice per group); and **l** sh-control and sh-CXCR4 PD/S-SCCs carrying mice (six mice per group). *, significant differences between the compared groups (*t*-test; *P* ≤ 0.05)

CXCR4 knockdown (Supplementary Fig. 6F–6H) or AMD3100 treatment blocked the proliferation of hSCC11 PD/S cells upon basal conditions (Fig. 7a, b). Similarly, abrogation of SDF-1 decreased hSCC11 cell proliferation (Fig. 7c). These findings indicate that an autocrine activation of SDF-1/CXCR4 signaling promotes proliferation of human PD/S-SCCs cells. In accordance with previous findings in mouse PD/S cells [11], no effect on hSCC11 cell proliferation was observed upon PDGFR inhibition by Imatinib treatment (Fig. 7d). However, PDGFR and CXCR4 inhibition significantly reduced the invasion capability of hSCC11 PD/S cells (Fig. 7e, f), indicating that activation of both signaling pathways promotes tumor cell invasion in advanced SCCs.

To determine the impact of PDGFR/SDF-1 signaling in human PD/S-SCC growth and metastasis, hSCC11 PD/S cells were engrafted in immunodeficient mice and when tumor growth was detected, mice were treated with Imatinib or AMD3100. In addition, sh-control, sh-CXCR4, and sh-SDF-1 hSCC11 PD/S cells were engrafted in immunodeficient mice. We found that PDGFR signaling inhibition, SDF-1 abrogation, or CXCR4 inhibition by RNA interference or AMD3100 treatment did not affect human PD/S-SCC growth (Fig. 7g–i; Supplementary Fig. 6I). However, inhibition of both PDGFR and SDF-1/CXCR4 signaling pathways significantly reduced the number and size of metastatic lesions developed in the lungs (Fig. 7j–l; Supplementary Fig. 6J).

Together, these results indicate that, similarly to what is described here for mouse advanced SCCs, PDGFR signaling promotes tumor cell invasion and metastasis in human advanced SCCs, which is mediated by the activation of SDF-1 signaling.

## Discussion

High risk and metastatic skin SCCs are occasionally treated with radiotherapy or conventional chemotherapy, but these treatments yield little clinical benefit [6]. Since long-term SCC growth is sustained by CSCs [7, 40], it is important to determine mechanisms controlling the proliferation and dissemination of this subset of tumor cells to block the aggressive growth and metastasis associated with advanced SCCs. Although previous studies have demonstrated that SDF-1 signaling promotes tumor growth and metastasis in other cancer types [22–24], the relevance of this signaling pathway in skin SCCs as well as the mechanisms regulating the pathway have been unknown. Here, we demonstrate that PDGFR signaling induces SDF-1 expression and the autocrine activation of SDF-1/CXCR4 signaling in cancer cells and L-CSCs, which promotes the invasion capability of these cells and lung metastasis in mouse and human PD/S-SCCs.

**Fig. 8:**
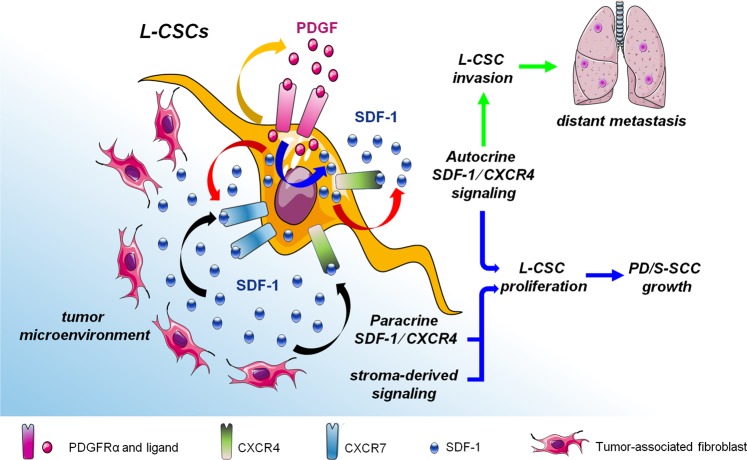
Impact of PDGFR/SDF-1 signaling crosstalk on PD/S-SCC growth and distant metastasis. Our results indicate that autocrine PDGFRα signaling (orange arrow) induces the expression of SDF-1 (blue arrow) and the autocrine activation of CXCR4/CXCR7 signaling in L-CSCs (red arrows). Fibroblast and stroma-derived SDF-1 can also activate this signaling pathway by a paracrine way (black arrows). Autocrine SDF-1/CXCR4 signaling promotes L-CSC invasion, promoting distant metastasis development. PD/S-SCCs growth may be promoted by the activation of SDF-1/CXCR4 pathway through autocrine or paracrine mechanisms, but also by alternative pathways, which can be activated by stroma-derived factors in response to PDGFR/SDF-1 signaling inhibition. This model shows mouse SCC findings, but can be also applied in human advanced SCCs

SDF-1 is expressed by stromal fibroblasts in mouse WD-SCCs and PD/S-SCCs, and its expression is significantly induced in CSCs of advanced SCCs. More than 50% of L-CSCs express CXCR7, whereas a subset of these cells expresses both CXCR4 and CXCR7 receptors. Therefore, SDF-1 signaling may be activated in these CXCR4/CXCR7-expressing CSCs not only by paracrine mechanisms, through SDF-1 secreted by the stroma fibroblasts, but also by autocrine mechanisms. We found that inhibition of SDF-1 and CXCR4 in L-CSCs blocks the in vitro proliferation of these cells upon basal conditions, indicating that autocrine activation of SDF-1 signaling promotes L-CSCs proliferation/survival, as previously reported in glioblastoma CSCs [23, 41]. CXCR4 knockdown in L-CSCs significantly reduced PD/S-SCC growth in immunodeficient mice, but not upon a proficient immune system, suggesting that impact of CXCR4 inhibition on tumor growth may be dependent of tumor microenvironment. In addition, abrogation of SDF-1 in L-CSCs had non-effect on in vivo tumor growth. One possible explanation is that stroma-derived SDF-1 drives tumor growth through CXCR7 and/or the remaining CXCR4 receptors of L-CSCs in SDF-1-interfered tumors. Alternatively, under the strong reduction of tumor cell-derived SDF-1, other cytokines or growth factors, produced by stromal cells, may act promoting cancer cell proliferation/survival and the growth of SDF-1 interfered PD/S-SCCs. Therefore, our results reveal a complex system of tumor growth regulation, implicating not only autocrine and paracrine SDF-1 signaling in tumor cells, but also other pathways probably activated by stroma-derived signals (Fig. 8).

It was proposed that constitutive expression of chemokines in metastasis target tissues is essential for driving metastasis, as CXCR4-expressing cells can home to tissues secreting high levels of SDF-1, such as lymph nodes, bone marrow, or lung [12]. Here, we demonstrated that SDF-1 knockdown or CXCR4 inhibition significantly reduced the in vitro invasion capability of L-CSCs and dramatically reduced the development of lung metastasis, independently of the immune state of mice. Therefore, autocrine SDF-1/CXCR4 signaling is necessary to facilitate L-CSC migration and invasion and to promote distant metastasis (Fig. 8). Accordingly, autocrine activation of the SDF-1 pathway was reported in various aggressive tumor types with enhanced distant metastasis capability [42, 43]. In addition, ectopic expression of CXCR4 in low metastatic HNSCC cells enables these cells to metastasize to regional lymph nodes, but not to the distant organs [44], whereas the ectopic overexpression of SDF-1 in CXCR4^+^ HNSCC and breast cancer cells enhanced in vitro cell motility and metastasis in lung [45, 46]. The autocrine activation of SDF-1 signaling described here could be essential for maintaining CXCR4 expression in L-CSCs, although in this scenario the directional homing of these migrating cells to SDF-1-expressing tissues may be disrupted. Furthermore, as SDF-1/CXCR4 signaling induces tumor cell survival (reviewed in [47]), autocrine activation of this signaling may promote migrating cell survival during dissemination or sustain the proliferation of these cells at the distant tissues.

Previous reports described a functional crosstalk between PDGFR and CXCR4 signaling, as activation of PDGFR signaling promotes stability and phosphorylation of CXCR4 in medulloblastoma cells, inducing SDF-1/CXCR4 signaling [48, 49]. We found that PDGFRα signaling induces SDF-1 expression, which in turn regulates the expression of PDGFRα. It is important to highlight that normal keratinocytes and E-CSCs express PDGF ligands, but not PDGFR or SDF-1, which are restricted to stroma cells [36, 50, 51]. In contrast, L-CSCs acquire the expression of PDGFRα, allowing not only the autocrine activation of this pathway [11], but also the expression of SDF-1 and the activation of SDF-1/CXCR4 signaling, which promote L-CSC invasion and metastasis. Furthermore, we observed that L-CSC invasion blockage induced by Imatinib was overridden by the ectopic expression of SDF-1, indicating that SDF-1 mediates PDGFRα-induced invasion and metastasis. Although inhibition of PDGFRα reduces *Sdf1* expression, no significant alterations in tumor cell proliferation or tumor growth were observed following pharmacological or genetic inhibition of PDGFRα in PD/S-SCCs [11], in agreement with that described after SDF-1 knocking-down. Therefore, we suggest that other factors or pathways support tumor growth, but not metastasis, in the absence of PDGFRα/SDF-1 signaling activity.

A similar functional crosstalk between PDGFR and SDF-1 was observed in human advanced SCCs. Strong induction of PDGFRα/β, as well as SDF-1 and CXCR4, was detected in tumor cells of patient PD/S-SCCs, and the inhibition of PDGFR signaling downregulated SDF-1 expression and vice versa. An autocrine activation of SDF-1/CXCR4 signaling promoted the in vitro proliferation and survival of these cells, although the inhibition of this signaling pathway did not affect human PD/S-SCC growth, following a similar pattern to that here described in mouse PD/S-SCCs growing in immunocompetent mice. However, the inhibition of PDGFR and SDF-1/CXCR4 signaling in human PD/S cells significantly reduced the invasive capacity of these cells and lung metastases development.

Together, our results show that a functional crosstalk between PDGFR/SDF-1 is induced in tumor cells at late stages of mouse and human skin SCC progression, in order to induce autocrine activation of SDF-1/CXCR4 signaling and to promote tumor cell invasion and metastasis. Therefore, the inhibition of PDGFR or/and SDF-1/CXCR4 pathways by currently available drugs, such as Imatinib or AMD3100, may be a possible therapeutic strategy for blocking metastasis development in patients with advanced human skin SCCs.

## Material and methods

### Mouse models and lineages of skin SCC generation

To generate the different lineages of skin SCC progression, small pieces (2–4 mm^3^) of spontaneous or DMBA/TPA-induced tumor that were developed in K14-HPV16^Tg/+^ mice (FVB/C57/Bl6 F1) [52] were engrafted in the back skin of 6-week-old male nude mice (Athymic Nude-Foxn1^nu^; Harlan Laboratories). Each orthotopic tumor (OT-SCC) was serially engrafted in a new immunodeficient mouse, as previously described [11]. Animal housing, handling, and all procedures involving mice were approved by the Bellvitge Biomedical Research Institute (IDIBELL) ethics committee, in accordance with Spanish national regulations.

### Human skin SCC samples

Samples of human skin SCCs were supplied by the Plastic Surgery and Pathology Units of the Hospital Universitario de Bellvitge (IDIBELL) and the Spanish Hospital Platform Biobank Network (RetBioH; www.redbiobancos.es). The protocol of sample collection was supervised and approved by the Ethical Committee of Clinical Research of Hospital Universitario de Bellvitge (IDIBELL). All patients were informed beforehand and their signed consent to participate was obtained.

### SCC cell isolation and flow cytometry analysis

Tumor cells from skin SCCs were analyzed by flow cytometry, and specific cell populations were isolated by FACS-sorter, as described in Supplementary Methods.

### Cell cultures

Tumor cells (hematopoietic lineage and endothelial-negative cells) isolated from mouse WD-SCCs (WD cells) and PD/S-SCCs (PD/S cells) from different lineages, and tumor cells isolated from human PD/S-SCCs (hSCC11 cells) (see Supplementary Methods for detailed protocol) were grown in DMEM-F12 medium (GIBCO Life Technologies) with 1× B27 (GIBCO Life Technologies) and penicillin/streptomycin (PAA Laboratories) (basic medium). Tumor cells isolated from human WD/MD-SCCs (hSCC24 cells) were grown in basic medium supplemented with EGF (20 ng/mL; Sigma). Cells were cultured at 37 °C in a humidified, 5% CO_2_ incubator. Protocols for analyzing cell proliferation and for cell transduction are described in detail in the Supplementary Methods.

### In vitro invasion assay

To test the invasion capability of human and mouse tumor cells in response to inhibitor treatments, CIM-16 plates (ACEA Biosciences) coated with 5% Matrigel (Factor-Reduced; BD Biosciences) were used. PD/S cells were previously treated without (control) or with AMD3100 (1 and 5 μg/mL; Chemscene LLC) and Imatinib (4 μM; LC Laboratories) for 48 h, and cells (8 × 10^5^, 4–6 replicates for sample) were seeded in the top chamber, and placed in the xCeLLigence system, maintaining the same concentration of inhibitors in the lower chamber. Each assay was performed twice. The cell index represents the cell invasion capacity.

### ELISA assay

To carry out these analyses, 1.5 × 10^6^ of mouse and human WD and PD/S cells were incubated for 48 h at 37 °C in basic medium. The medium was then collected and centrifuged (13,000 r.p.m.) for 10 min and SDF-1 concentration in the supernatant was analyzed using the PeproTech ELISA kit (Mini ABTS ELISA Development kit) (three replicates per sample), following the manufacturer’s recommendations. Substrate solution (TMB Liquid Substrate) was added and incubated for 20 min at room temperature. The absorbance was measured at 450 nm.

### Tumor cell grafting and in vivo treatments

*Sdf1*, *Cxcr4,* and *Cxcr7* expression and tumor cell populations expressing these receptors were analyzed in skin SCCs spontaneously developed in male and female K14-HPV16^Tg/+^ mice (four samples per group), and in WD-SCCs and PD/S-SCCs generated after subcutaneous engrafting WD cells (1 × 10^6^ cells) and PD/S cells (1 × 10^4^ cells) from OT7 and OT14 lineages with Matrigel (1:1; BD Biosciences) in syngeneic immunocompetent mice (6-week-old C57Bl6/FVB F1 male mice; four samples for group). The impact of CXCR4, CXCR7, SDF1 knockdown on mouse tumor growth and metastasis was studied by subcutaneously co-injecting 4 × 10^3^ sh-control (*n* = 10), sh-CXCR4 (*n* = 11), sh-CXCR7 (*n* = 11), and sh-SDF1 (*n* = 11) mouse PD/S cells with Matrigel (1:1; BD Biosciences) in 6-week-old male nude mice. Following the same protocol, 4 × 10^3^ sh-control (*n* = 6) and sh-CXCR4 (*n* = 10) PD/S cells were injected in immunocompetent mice. To test the in vivo effect of CXCR4 and SDF1 knockdown on human PD/S-SCC growth and metastasis, 1 × 10^6^ of sh-control (*n* = 15), sh-CXCR4 (*n* = 10), and sh-SDF1 (*n* = 5) hSCC11 cells were engrafted in NOD-*scid* IL2Rg^null^ (NSG) mice. Tumor growth was monitored and tumors were excised after 25–27 days (mouse SCCs) or 30–32 days (human SCCs), when they reached a critical size. Mice were sacrificed 20 days (mouse PD/S-SCCs) or 10 days (human PD/S-SCCs) after tumor resection. The lungs were recovered and formalin-fixed to quantify the metastatic lesions (5–6 samples per group).

For pharmacological inhibition of CXCR4 in mouse PD/S-SCCs, 4 × 10^3^ OT14 PD/S cells were subcutaneously engrafted in syngeneic immunocompetent mice. For pharmacological inhibition of PDGFR and CXCR4 in human PD/S-SCCs, 1 × 10^6^ of hSCC11 cells were engrafted in NSG mice. When the tumors were palpable, mice were randomly distributed between control and treated groups, in order that tumor size was similar in both groups at the start of the treatment. Then, mice were intraperitoneally treated with AMD3100 (Glentham Life Sciences; 15 mg/kg; diluted in sterile serum), or serum (*n* = 7 tumors per group for mouse and human SCCs), or orally administered with Imatinib (LC Laboratories; 150 mg/kg; diluted in water) or water (*n* = 9 tumors per group), daily. Tumors were excised when they reached a critical size (20 days post-engraftment) and mice continued to be treated until sacrifice, 10–15 days (for human SCCs) or 20 days (for mouse SCCs) after tumor resection. Then, lungs were recovered and formalin-fixed to quantify the development of the metastatic lesions (5–6 samples per group). Tumors that did not grow after 4–5 weeks of the engraftment or mice that died before the experiment endpoint were excluded.

### Histology, immunohistochemistry, and western blot assays

Tumor and lung samples were fixed in 4% formaldehyde overnight at 4 °C, paraffin-embedded, and sectioned at 4 μm. For histopathological analysis and quantification of metastatic lesions, tumor and lung sections were stained with hematoxylin and eosin. Protocols for immunofluorescence assays and western blot analysis are described in detail in the Supplementary Methods.

### Reverse transcription and quantitative real-time PCR

Total RNA was extracted from tumors and cells using Trizol Reagent (Invitrogen). Reverse transcription reactions and quantitative real-time PCR were carried out as described in Supplementary Methods.

## Supplementary information

Supplementary Information.

Supplementary Figures.
