# Supplementary material for: PDGFR-induced autocrine SDF-1 signaling in cancer cells promotes metastasis in advanced skin carcinoma
Source: Oncogene. 2019 Mar 15;38(25):5021–37. doi: 10.1038/s41388-019-0773-y (PMC6756210; doi:10.1038/s41388-019-0773-y)
Supplement: Supplementary file 1 — Supplementary Information. [file 41388_2019_773_MOESM1_ESM.pdf]

SUPPLEMENTARY INFORMATION

- **Supplementary Figures 1 through 6:**

Figure 1. SDF-1, CXCR4 and CXCR7 expression in advanced SCCs developed in immunodeficient and immunocompetent mice.

Figure 2. Effect of CXCR4 inhibition and SDF-1 knockdown on PD/S cell proliferation.

Figure 3. Impact of CXCR4 and CXCR7 knockdown on L-CSC proliferation and on the frequency of CXCR4- and CXCR7-expressing CSC population of PD/S-SCCs generated by these interfered cells.

Figure 4. Effect of SDF-1 abrogation in PD/S cells on EMT marker expression, tumor growth and metastasis development.

Figure 5. Analysis of the impact of alterations in *Sdf1* expression on PDGFRα expression.

Figure 6. Effect of CXCR4 inhibition on human advanced SCC growth and metastasis.

- Supplementary Figure Legends: Legends of Supplementary Figures 1-6.

- Supplementary Table:

Table 1. Features of patient skin SCC samples.

Table 2. Antibodies used in flow cytometry, immunohistochemistry, immunoflorescence and western blot analysis.

Table 3. Primers used in qRT-PCR.

- Supplementary Material and Methods: Include a more detailed description of Material and Methods.

- Supplementary References

SUPPLEMENTARY FIGURE LEGENDS

Supplementary Figure 1. Induction of SDF-1 and CXCR4 expression in tumor cells of PD/S-SCCs of immunodeficient and immunocompetent mice.

A and **B**, Representative images of the immunodetection of CXCR4 and CXCR7 in sections of mouse WD-SCCs and PD/S-SCCs that were developed in immunodeficient mice. Scale bar, 40 μm.

C and **D**, mean (± SE) mRNA levels of the indicated genes, relative to *Gapdh*, in WD-SCCs and PD/S-SCCs, which were generated (**C**) spontaneously in the skin of K14-HPV16$^{Tg/+}$ mice (four tumor samples per group), and (**D**) after the engraftment of WD and PD/S cells of the indicated lineages in syngeneic immunocompetent mice (four samples per tumor type and lineage).

E, mean (± SE) levels of *Sdf1* mRNA, relative to *Gapdh*, as quantified by qRT-PCR, in tumor cells (α6-integrin^{+}/CD45^{-}/CD31^{-} cells), immune cells (α6-integrin^{-}/EpCAM^{-}/CD45^{+}/CD31^{-} cells) and fibroblasts (α6-integrin^{-}/EpCAM^{-}/CD45^{-}/CD31^{-} cells) isolated from WD-SCCs and PD/S-SCCs developed in immunocompetent mice (2-3 samples per group) by FACS-sorter.

F, mean percentage (± SE) of α6-integrin^{+}/CXCR4^{+} and α6-integrin^{+}/CXCR7^{+} cells in the indicated tumors (four tumor samples per group), as quantified by flow cytometry (**G**).

G, representative results of the quantification by flow cytometry of the percentage of α6-integrin^{+}/CXCR4^{+} and α6-integrin^{+}/CXCR7^{+} cells (indicated in each panel) in WD-SCCs and PD/S-SCCs developed in immunocompetent mice.

H, mean percentage (± SE) of the indicated cell populations in OT14 WD-SCCs and PD/S-SCCs developed in immunocompetent mice (four tumor samples per group).

*, significant differences between the compared groups (t-test; $P \leq 0.05$).

Supplementary Figure 2. SDF-1 abrogation and inhibition of CXCR4 reduces the proliferation/survival of L-CSCs.

A-C, quantification by flow cytometry of the percentage of indicated cell populations in tumor cells isolated from PD/S-SCCs (PD/S cells; three independent cell cultures) and maintained in culture, which are enriched in α6-integrin^{+}/CD34^{+} CSCs.

D, cell proliferation upon SDF-1α and/or AMD3100 treatment, as measured by MTT. Mean (± SE) arbitrary units of fluorescence (a.u.f.) in treated cells relative to cells growing in basal conditions.

E, quantification by ELISA assays of the SDF-1 concentration in the culture medium of PD/S cells transduced with sh-control or the indicated sh-SDF-1 lentivirus constructions (two samples per group).

F, representative proliferation kinetics (mean ± SE of a.u.f. relative to day 0) of PD/S cells transduced with sh-control and sh-SDF1.1 construct, as measured by MTT.

G, mRNA levels (mean ± SE; two samples of cell cultures per lineage) of the indicated genes in sh-SDF-1 PD/S cells, relative to control (sh-control) cells.

*, significant differences between the compared groups (t-test; $P \leq 0.05$).

Supplementary Figure 3. Abrogation of CXCR4 in L-CSCs reduces the growth of PD/S-SCC developed in immunodeficient mice.

A, *Cxcr4* and *Cxcr7* mRNA levels (mean ± SE), relative to *Gapdh* in PD/S cells transduced with sh-control or different CXCR4 and CXCR7 sh-RNA lentivirus constructions.

B, mean mRNA levels (± SE; three samples of cell cultures per lineage) of the indicated genes in sh-CXCR4.3 (transduced with sh-RNA number 3) and sh-CXCR7.1 (transduced with sh-RNA number 1) PD/S cells, relative to control (sh-control) cells.

C, representative proliferation kinetics (mean ± SE of a.u.f. relative to day 0) of sh-control, sh-CXCR4.3 and sh-CXCR7.1 PD/S cells, as measured by MTT.

D and **E**, mean percentage (± SE) of the indicated cell populations in sh-control, sh-CXCR4.3 and sh-CXCR7.1 PD/S-SCCs (6-10 tumor samples per group), as measured by flow cytometry.

F, representative results of the quantification by flow cytometry of the percentage of CXCR4- and CXCR7-expressing α6-integrin$^+$/CD34$^+$ CSCs in the indicated PD/S-SCCs.

G, mean percentage of proliferating cells (± SE; 3-4 tumor samples per group), as determined in (**H**).

H, representative images of the immunodetection of phosphorylated H3 (Ser10) in the indicated PD/S-SCCs developed in immunodeficient mice. Scale bar, 20 μm.

I, mean (± SE) mRNA levels of the indicated genes in tumor cells isolated from sh-CXCR4 PD/S-SCCs relative to cells isolated from sh-control tumors generated in immunocompetent (3-4 samples per group).

J, mean (± SE) of *Vegfr2* mRNA levels, relative to *Gapdh* in the indicated PD/S-SCCs (six tumor samples per group).

*, significant differences between the compared groups (t-test; $P \leq 0.05$).

Supplementary Figure 4. SDF-1 abrogation in L-CSCs blocks lung metastasis development without altering PD/S-SCC growth.

A, growth kinetics (mean ± SE of tumor size, mm^3) of control (sh-control) and SDF-1 knocked-down (sh-SDF-1.1) PD/S-SCCs (eleven tumors per group).

B, mean (± SE) *Sdf1* mRNA levels in tumor cells isolated from sh-SDF-1.1 PD/S-SCCs, relative to sh-control tumor cells (four tumor cell populations per group).

C and **D**, representative results of the quantification by flow cytometry of the indicated cell populations in sh-control and sh-SDF-1.1 PD/S-SCCs.

E, mean frequency of metastatic foci per lung section (± SE, categorized by size, mm^2) in mice carrying SDF-1 expressing (sh-control) and SDF-1 knocked-down (sh-SDF-1.1) tumors (six mice per group).

F, mean (± SE) mRNA levels of the indicated genes in tumor cells isolated from sh-SDF-1.1 PD/S-SCCs, relative to sh-control tumor cells (four tumor cell samples per group).

*, significant differences between the compared groups (t-test; $P \leq 0.05$).

Supplementary Figure 5. SDF-1 abrogation induces a down-regulation of PDGFRα expression

A, mean (± SE) *Pdgfra* mRNA levels in sh-SDF-1.1 PD/S cells relative to sh-control cells, as measured by qRT-PCR.

B, PDGFRα expression in control and SDF-1-interfered PD/S cells. GAPDH was used as a protein-loading control.

C, quantification by ELISA assays of SDF-1 concentration (mean ± SE) in the culture medium of two independent populations of control or SDF-1 overexpressing PD/S cells.

*, significant differences between the compared groups (t-test; $P \leq 0.05$).

Supplementary Figure 6. Inhibition of CXCR4 blocks distant metastasis in human advanced SCCs.

A, Representative images of the immunodetection of CXCR4 in patient samples of WD/MD-SCCs and PD/S-SCCs. Scale bar, 60 μm.

B, tumor cells isolated from a patient WD-SCC (hSCC24 cells) grow *in vitro* as adherent cells, exhibiting a typical epithelial morphology, whereas tumor cells isolated from a PD/S-SCC (hSCC11 cells) grow in suspension, forming spheres.

C, quantification by flow cytometry of the percentage of tumor cells expressing α6-integrin and EpCAM epithelial markers in the indicated cell cultures.

D, mean (± SE) levels of mRNA of *CDH1*, *Vimentin* and EMT-inducer transcription factors, relative to *GAPDH* in the indicated cell cultures.

E, representative images of the PDGFRα and PDGFRβ expression in tumor cells from human WD-SCCs and PD/S-SCCs. GAPDH was used as a protein-loading control.

F, mean (± SE) levels of *CXCR4* mRNA in hSCC11 PD/S cells transduced with different CXCR4 sh-RNA lentivirus constructions relative to sh-control cells, as measured by qRT-PCR (one sample per group).

G, representative results of the quantification by flow cytometry of α6-integrin$^+$/CXCR4$^+$ cells in control (sh-control) and in the indicated CXCR4 knocked-down (sh-CXCR4) hSCC11 cells.

H, mean percentage (± SE) of α6-integrin$^+$/CXCR4$^+$ cells in control (sh-control) and CXCR4 knocked-down (sh-CXCR4.6) PD/S-SCCs (seven tumor samples per group), as quantified by flow cytometry.

I, growth kinetics (mean ± SE of tumor size, mm^3) of tumors generated after hSCC11 cell engrafting in immunodeficient mice, which were treated with vehicle solution (control) and AMD3100 (seven tumors per group). *P* value (t-test) of the compared groups is indicated.

J, mean of metastatic foci (± SE) per lung section (categorized by size, mm^2) developed in control and AMD3100-treated mice (six mice per group).

*, significant differences between the compared groups (t-test; $P \leq 0.05$).

		% of indicated tumor region in overall sample*		mRNA/GAPDH	
Sample	***Classification /Grade***	***% MD-SCC***	***% PD-SCC / % spindle SCC***	***SDF1***	***CXCR4***
T1	PD-SCC / G3	0%	95% / 5%	163.5	59.9
T2	PD-SCC / G3	0%	40% / 60%	62.7	184.2
T5	PD-SCC/ G3	55%	45% / 0%	24.1	84.1
T6	MD-SCC / G2	100%	0% / 0%	16.7	24.9
T9	MD-SCC / G2	60%	0% / 0%	16.4	9.8
T10	PD-SCC / G3	55%	40% / 0%	0.97	4.6
T16	PD-SCC / G4	0%	0% / 100%	6.6	102.3
T23	MD-SCC / G2	90%	0% / 0%	8.2	13.3
T24	WD-SCC /G1	0%	0% / 0%	7.6	4.5
T26	PD-SCC / G3	0%	100% / 0%	51.8	93.4
T30	PD-SCC / G3	60%	40% / 0%	n.d.	30.3
T34	MD-SCC / G2	30%	0% / 0%	22.9	n.d.
T39	MD-SCC / G2	100%	0% / 0%	6.2	9.2
T40	MD/PS-SCC G2	30%	0% / 0%	12.9	6.3
T41	WD/MD-SCC / G2	50%	10% / 0%	0.5	5.3
T42	PD-SCC / G4	0%	0% / 100%	n.d.	44.7
T44	PD-SCC / G3	0%	100% / 0%	62.7	16.5
T45	MD-SCC / G2	85%	15% /0%	24.1	31.9
T46	MD-SCC / G2	100%	0% / 0%	16.7	23.9
T47	MD-SCC / G2	100%	0% / 0%	22.8	28.5
T48	PD-SCC / G3	55%	40%/ 0%	19.8	16.9
T49	PD-SCC / G4	0%	0% / 100%	33.8	42.0
T51	MD-SCC / G2	70%	0% / 0%	4.6	21.4
T52	MD/PD-SCC G2/G3	70%	30% / 0%	n.d.	17.6
T53	MD-SCC / G2	75%	5% / 0%	6.6	8.1
T54	MD-SCC / G2	90%	10% / 0%	6.2	7.0
T55	MD-SCC / G2	95%	5% / 0%	47.6	10.5
T56	PD-SCC / G3 (M)	0%	100% / 0%	28.0	24.2

Supplementary Table 1. Features of patient skin SCC samples. (*) Human skin SCCs frequently show intra-tumoral heterogeneity. The histopathological grade of different regions and the percentage represented by each region in the overall sample of each tumor were determined by a pathologist. (M) Metastasis, n.d., no data.

Antibody	Dilution	Source	Catalog number
Primary antibodies			
anti-CD34-eFluor 660	1/100	eBioscience	50-0341-82
α6-integrin(CD49f)-PE	1/50	BD Bioscience	555736
α6-integrin(CD49f)-FITC	1/20	BD Bioscience	555735
anti-CXCR4-APC	1/50	Milteny Biotec	130-102-913
anti-CXCR4-VIO 615	1/100	Milteny Biotec	130-107-610
anti-CXCR7-PE	1/75	BioLegend	331104
anti-EpCAM-FITC	1/350	Milteny Biotec	130-098-113
anti-CD45-PE	1/200	BD Bioscience	50-0451-U100
anti-CD31 (IF)	1/200	BD Bioscience	550274
anti-CD31 (FACS)	1/100	BD Bioscience	550274
anti-phosphorylated H3 (Ser10)	1/200	Cell Signaling	9706S
anti-CXCR4	1/100	Sigma	C8352
anti-CXCR7	1/100	Acris	AP17961PU-N
anti-SDF-1	1/100	R&D Systems	MAB350
anti-PDGFRα	1/500	R&D Systems	AF-307-SP
anti-PDGFRβ	1/1000	Santa Cruz Biotec	sc-374573
anti-GAPDH-HRP	1/6000	Cell Signaling	3683S
Secondary antibodies			
Dynabeads anti-rat	1/50	Invitrogen	11035
anti-rabbit-Alexa 488	1/400	ThermoFisher	A27034
anti-rat-Alexa 488	1/400	ThermoFisher	A11006
anti-rabbit-Alexa 568	1/600	ThermoFisher	A10042
anti-mouse EnVision-HRP	Ready-to-use	DAKO	K4063
anti-rabbit-HRP	1/1000	DAKO	P0448

Supplementary Table 2. Antibodies used in flow cytometry, immunohistochemistry, immunoflorescence and western blot analysis.

Gene	Forward (5' - 3')	Reverse (5' - 3')
MOUSE		
Gapdh	AGGTCGGTGTGAACGGATTTG	TGTAGACCATGTAGTTGAGGTC
Ppia	GTTCATGCCTTCTTTCACCTTCCC	CAAATGCTGGACCAAACACAAACG
Pdgfra	CAGTCCACCCGTGTGCT	GAAAATTCAACAGCAGCTGGT
Sdf1	GGCGTCTGACTCACACCTCT	AGTGTGCATTGACCCGAAAT
Cxcr4	CCATGGAACCGATCAGTGTG	TTTTCATCCCGGAAGCAGG
Cxcr7	CTGTCAGCTGGAGAATGTGC	TGCGGTTGATGAAGCTGTAG
Snail	CTTGTGTCTGCACGACCTGT	AGTGGGAGCAGGAGAATGG
Twist	AGCTACGCCTTCTCCGTCT	TCCTTCTCTGGAAACAATGACA
Vimentin	AGAGAGAGGAAGCCGAAAGC	TCCACTTTCCGTTCAAGGTC
Vegfr2	ACGGGAGACGTCCTTCATAA	GTGCCGACGAGGATAATGAC
HUMAN		
GAPDH	CAAGATCATCAGCAATGCCT	AGGGATGATGTTCTGGAGAG
HPRT	TGACACTGGCAAAACAATG	GGTCCTTTTCACCAGCAAG
GUSB	CTCATTTGGAATTTTGCCGATT	CCGAGTGAAGATCCCCTTTTTA
PDGFRA	GTCTGGAGCGTTTGGGAAGGT	GATCTGGCCGTGGGTTTTAGC
PDGFRB	GGACATACCCCCGCAAAGAA	CTAACTCGGCACTGGGGATGT
SDF1	TGTGCCCTTCAGATTGTAGCC	TCGAGTGGGTCTAGCGGAAA
CXCR4	GCAAGGCAGTCCATGTCATC	TTGGCCTCTGACTGTTGGTG
CXCR7	ACATGCCCAACAAAAGCGTC	ACCACGGAGTTGGCAATCAT
CDH1	CAGAATTGCTCACATTTCC	GGATTTGATCTGAACCAGGT
VIMENTIN	GGCTCAGATTCAGGAACAGC	GCTTCAACGGCAAAGTTCTC
TWIST	GGAGTCCGCAGTCTTACGAG	TCTGGAGGACCTGGTAGAGG
ZEB1	TTTGGCTGGATCACTTTCAAG	GCCAATAAGCAAACGATTCTG
ZEB2	TGTAGATGGTCCAGAAGAAATG	CCATTGTTAATTGCGGTCT

Supplementary Table 3. Primers used in qRT-PCR

SUPPLEMENTARY MATERIALS AND METHODS

Isolation of SCC cells

Excised mouse tumors and fresh human skin SCC samples were minced and incubated with collagenase I (60 U/ml; Sigma) and dispase (0.7 U/ml; Gibco) overnight at 37 ºC. Cell suspensions were filtered and depleted of red blood cells using ACK lysis buffer (Lonza). For endothelial cell depletion, mouse cell suspensions were incubated with anti-CD31 antibody for 30 min at 4 ºC, and then with Dynabeads® anti-rat for 30 min at 4 ºC. Isolated tumor cells were then plated and cultured as described in Methods.

Flow cytometry analysis and sorting

For flow cytometry analysis and sorting, isolated human and mouse tumor cells in blocking buffer (5% FBS in PBS) were incubated with anti-CD34-eFluor 660, PE-labeled α6-integrin (CD49f) antibody or FITC-labeled α6-integrin (CD49f) antibody, anti-CXCR4-APC or anti-CXCR4-VIO 615, anti-CXCR7-PE and anti-EpCAM-FITC antibody for 30 min at 4 ºC (Supplementary Table 2). In addition, isolated tumor cells were incubated for 30 min at 4ºC with anti-CD45-PE antibody, to exclude immune cells in flow cytometry assays or to isolate $CD45^+$ immune cells by FACS-sorter. Cells were then washed with 0.5% BSA, 2 mM EDTA in PBS, and resuspended in analysis buffer (2% FBS, 2 mM EDTA in PBS). Live cells excluded DAPI (Sigma) staining. FACS-sorting and analysis were carried out with BD Bioscience Aria and Fusion II equipment.

Cell proliferation assays and treatments

To analyze cell proliferation, PD/S cells were plated (2×10^3 mouse cells and 4×10^3 human cells per well) in six replicates on 96-well plates in basic medium, with a previous withdrawal of puromycin for 48 h in the case of transduced cells. To evaluate

the effect of AMD3100, 4 x 10^3 mouse and human cells were seeded as described above. Then, fresh basic medium without or with AMD3100 (1 μg/mL and 5 μg/mL; Chemscene LLC) or with murine or human SDF-1α (150 ng/mL; PeproTech) was added. To evaluate the effect of Imatinib on human hSCC11 cells, 4 x 10^3 cells were seeded per well in six replicates on 96-well plates in basic medium. Then, fresh basic medium without or with Imatinib (LC Laboratories) and without or with PDGF-AA (30 ng/ml), PDGF-BB (30 ng/ml) or PDGF-CC (100 ng/ml) was added. In these assays, fresh medium with factors and/or inhibitors was added every 3 days. Each assay was carried out 2-3 times. Cell proliferation/survival were measured by calculating the mean (± SE) absorbance at 560 nm after 6 days of treatment by using 3-(4,5-dimethyl-2-thiazolyl)-2,5-diphenyl-2H-tetrazolium bromide (MTT) assay.

Lentiviral and retroviral cell transductions

To knock down mouse CXCR4, CXCR7, SDF-1 and PDGFRα and human CXCR4 and SDF-1 expression, different shRNA lentivirus vectors (from Dharmacon for mouse and Sigma for human) were tested. To perform the *in vitro* and *in vivo* experiments the most interfering shRNA was used. For SDF1 overexpression, PD/S cells were transduced with pBabe SDF-1α retroviral vector (Addgene) or empty pBabe vector. To carry out cell transduction, lentivirus and retrovirus particles were produced in 293T cells using standard protocols. PD/S cells growing as spheres, were disaggregated by Accutase Solution (Sigma) for 10 min and 1 x 10^6 cells were incubated with 293T medium containing lentivirus particles, following standard infection protocols.

Histology, immunohistochemistry and immunoblotting assays

Tumors and lungs were fixed in 4% formaldehyde overnight at 4 ºC, paraffin-embedded and sectioned at 4 μm. For histopathological analysis and to quantify metastasis foci, tumor and lung sections were stained with hematoxylin and eosin, as previously described (1). For CXCR4, SDF-1 and phosphorylated H3 immunodetection, tumor paraffin-embedded sections were used and antigens were previously retrieved in 10 mM sodium citrate (pH 6.0). For CD31 and CXCR7 immunofluorescent staining, tumor cryosections were used. Tumor sections (3-4 samples for tumor group) were blocked with 5% horse serum or 5% BSA in PBS for 1 h at room temperature. The primary antibodies used were anti-phosphorylated H3 (Ser10), anti-CD31, anti-CXCR4, anti-CXCR7 or anti-SDF-1 antibodies (Supplementary Table 2). Tumor sections were then incubated with anti-rabbit or anti-rat Alexa-488 or Alexa-568 secondary antibodies for 1h at room temperature (Supplementary Table 2). Nuclei were stained with 4'6'-diamidino-2-phenylindole (DAPI). For SDF-1 immunodetection sections were incubated with secondary anti-mouse EnVision antibody followed by the DAB developing system (Dako). Samples were counterstained with hematoxylin and visualized under light microscopy. Nikon Eclipse 80i, Leica DM6000B, Leica TCS SP5 confocal, and Zeiss Apotome microscopes were used.

For western blot analysis, whole-cell extracts from tumor and culture cells were prepared in lysis buffer, containing 50 mM Tris pH 8, 5 mM EDTA, 350 mM NaCl, 0.5% NP-40, 10% glycerol, 1 mM phenylmethanesulfonylfluoride (PMSF), 2 mM NaF, 0.1 mM Na_3VO_4, 1 mM DTT, PhosSTOP (Roche), Complete (Roche), 0.1% SDS (Invitrogen) and 1% Triton X-100 (Thermo Scientific), as previously described (2). After transfer, proteins were incubated overnight with the antibodies anti-PDGFRα; anti-PDGFRβ and anti-GAPDH-HRP (Supplementary Table 2). Antibody binding was

detected with a secondary antibody coupled to horseradish peroxidase using enhanced chemiluminescence detection reagents (Amersham).

Reverse transcription and quantitative PCR

RNA samples were previously treated with DNAse (Sigma). Reverse-transcription reactions were carried out with the High Capacity cDNA Reverse Transcription kit (Applied Biosystems). Real-time PCR reactions were performed (three replicates for each sample) on an Applied QuantStudio5, using SYBR Green Mix (Applied Biosystems) and primers described in Supplementary Table 3. *Gapdh/GAPDH, Ppia, HPRT* and *GUSB* were used to normalize the gene expression for all human and mouse samples. mRNA levels were shown as relative to *Gapdh/GAPDH* mRNA, or alternatively as fold change. In this case, mean of mRNA levels relative to two or three housekeepings was calculated.

Statistical analysis

Statistical analysis was performed using Prism 5.0 Software (GraphPad software, San Diego, CA). Normal distribution of data and similarity of variances between compared groups were checked using the Shapiro-Wilk Normality test and F-Fisher test, respectively. Student's *t*-test (two-tailed) was used to determine the significance of differences between groups, as detailed in legend figures. Data are presented as the means ± SE. Scatter plot and Pearson coefficient were to correlate the relative gene expression of *CXCL12* and *PDFGRA/B* in patient samples. No statistical methods were used to predetermine sample size in *in vivo* experiments, which was estimated based on our previous experience and similar experiments reported in literature.

SUPPLEMENTARY REFERENCES

1. da Silva-Diz V, Sole-Sanchez S, Valdes-Gutierrez A, *et al.* Progeny of Lgr5-expressing hair follicle stem cell contributes to papillomavirus-induced tumor development in epidermis. *Oncogene* 2013;**32**(32):3732-43.

2. da Silva-Diz V, Simon-Extremera P, Bernat-Peguera A, *et al.* Cancer Stem-like Cells Act via Distinct Signaling Pathways in Promoting Late Stages of Malignant Progression. *Cancer Res* 2016;**76**(5):1245-59.
