# Supplementary figures and images for: PDGFR-induced autocrine SDF-1 signaling in cancer cells promotes metastasis in advanced skin carcinoma
Source: Oncogene. 2019 Mar 15;38(25):5021–37. doi: 10.1038/s41388-019-0773-y (PMC6756210; doi:10.1038/s41388-019-0773-y)

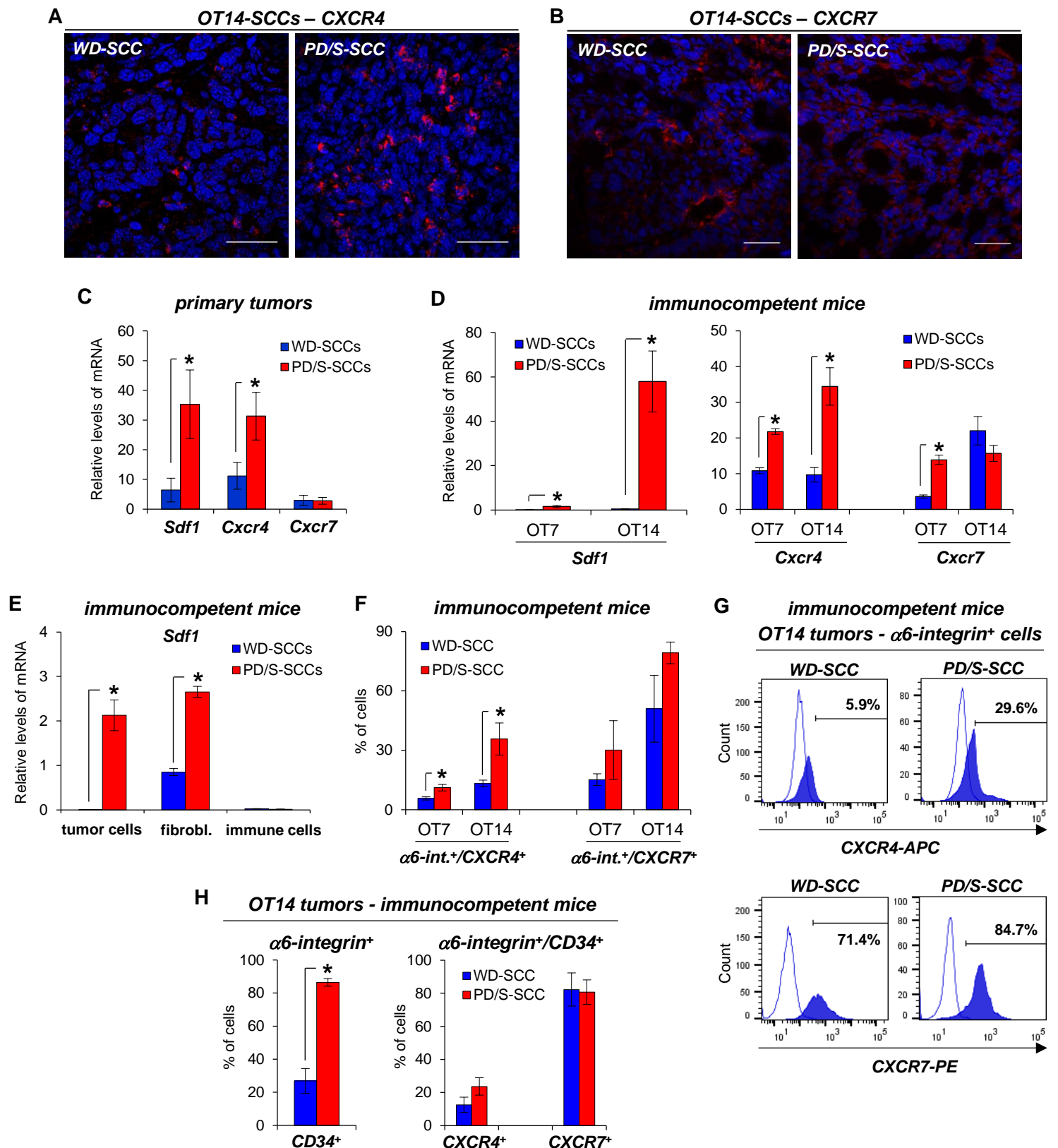

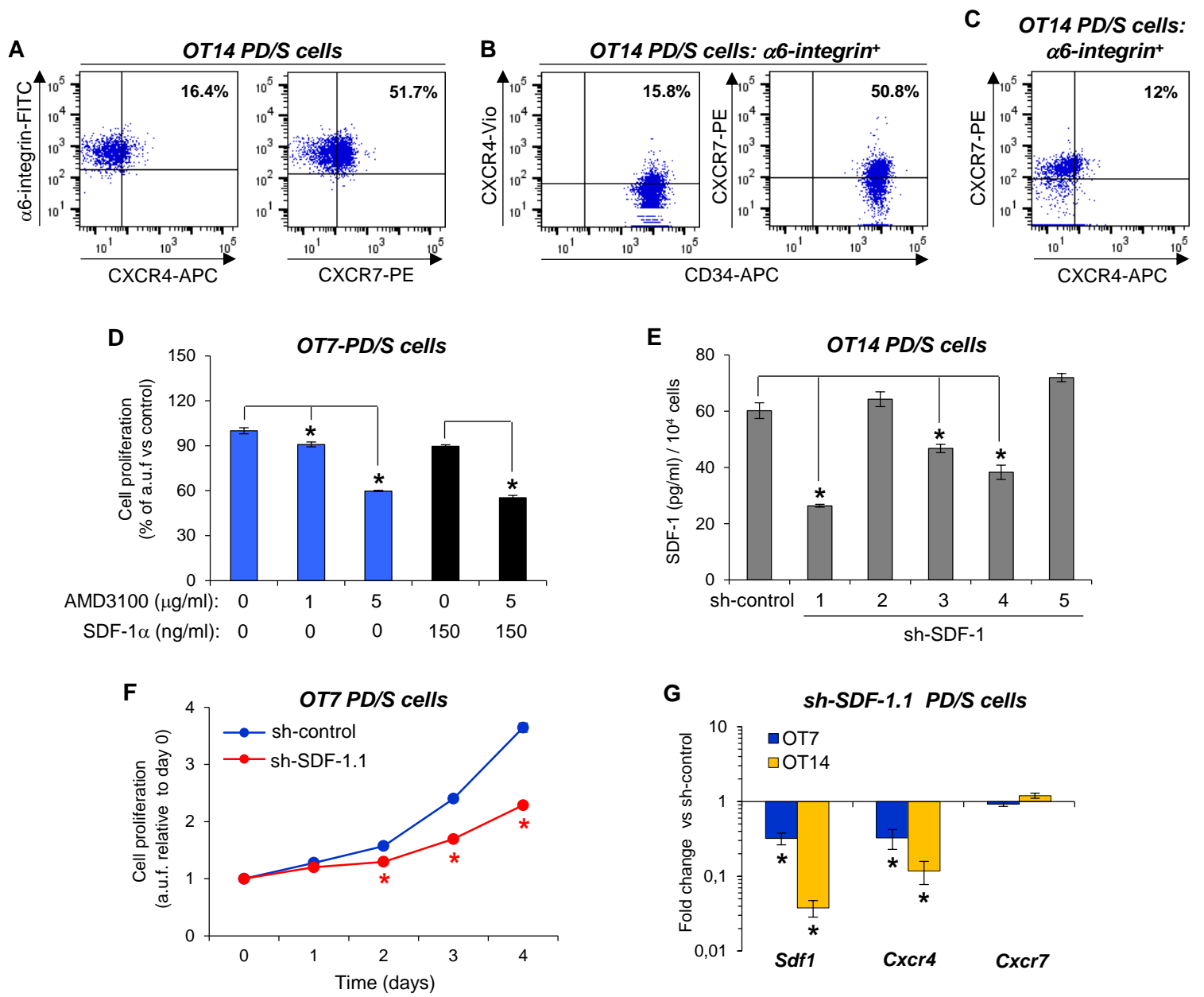

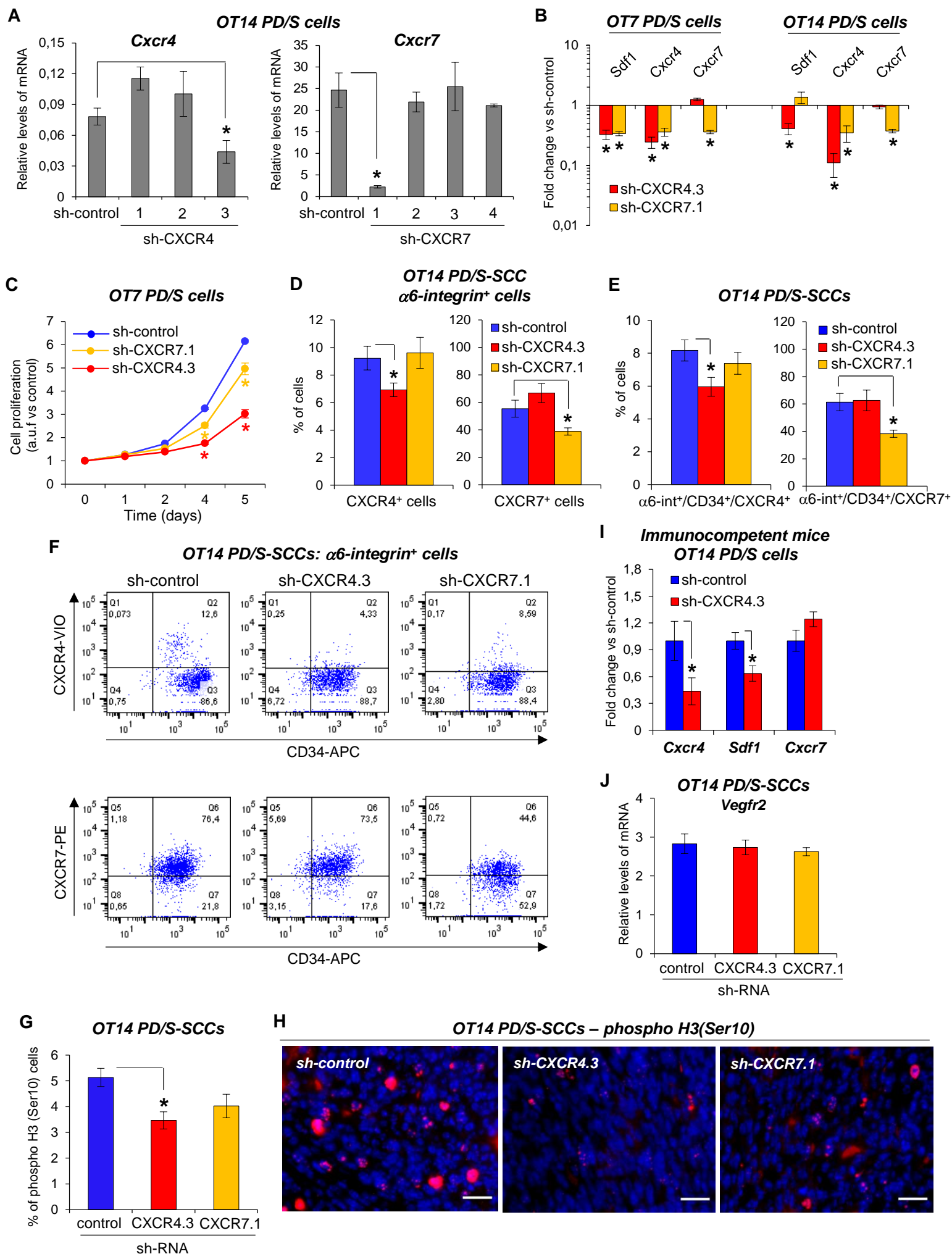

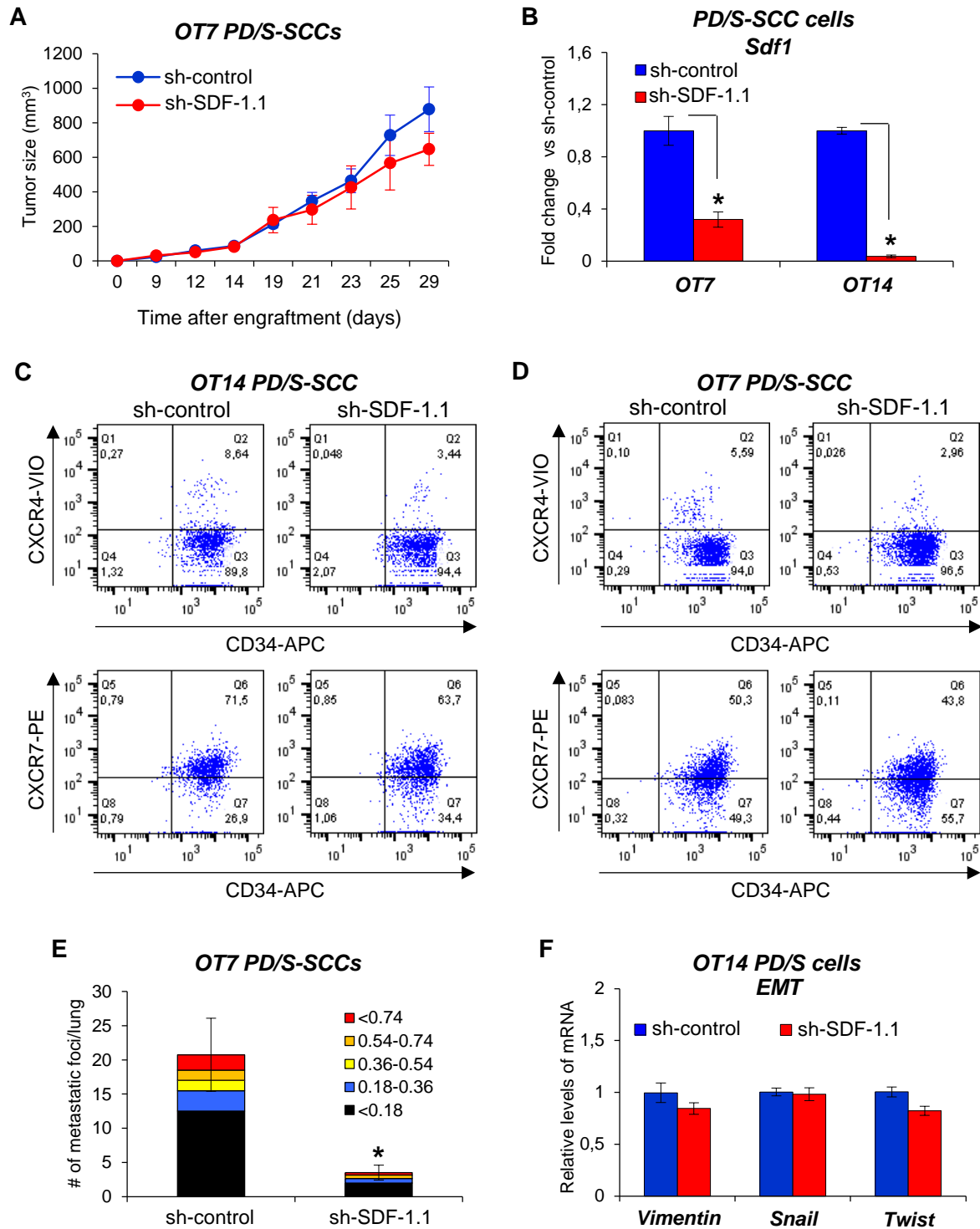

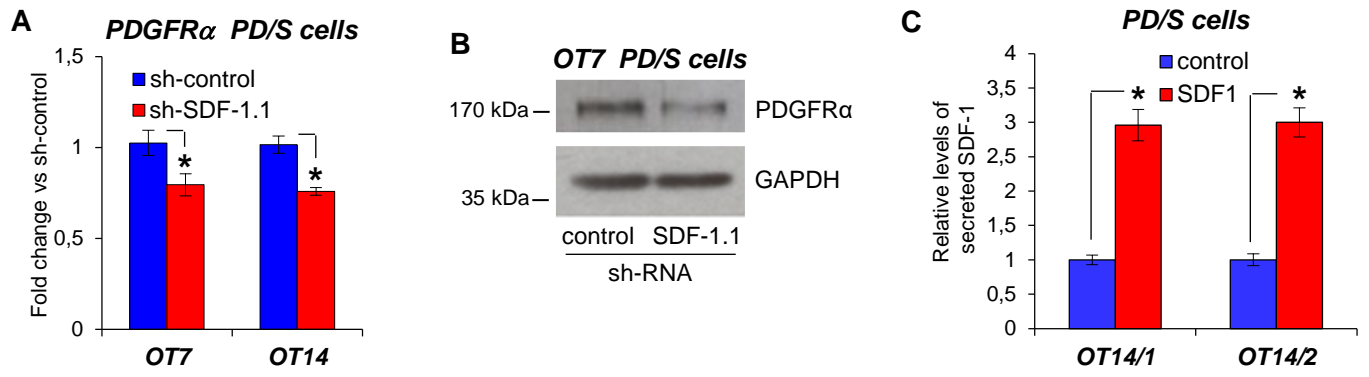

# Primary human SCCs – CXCR4

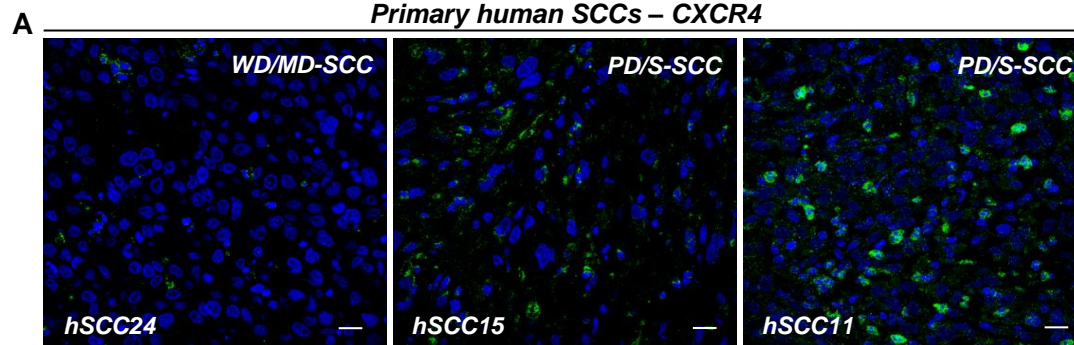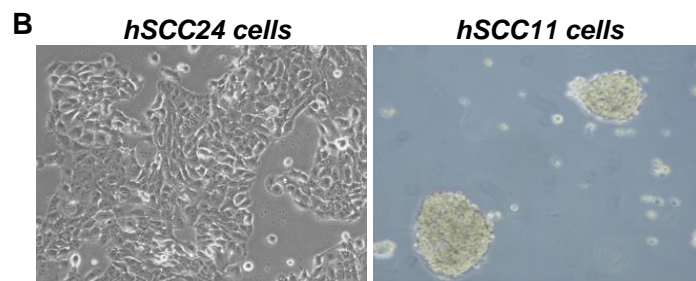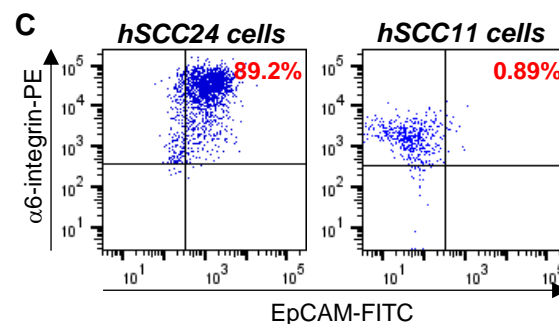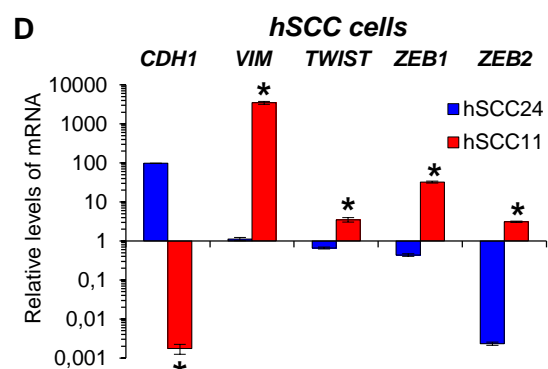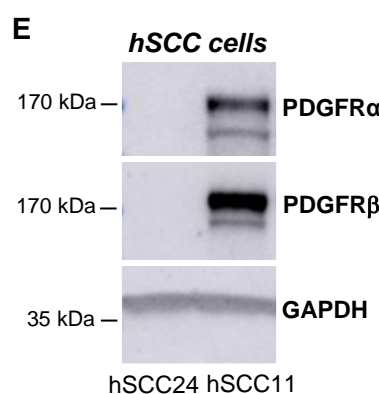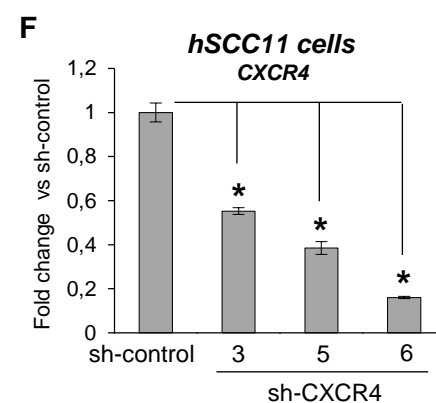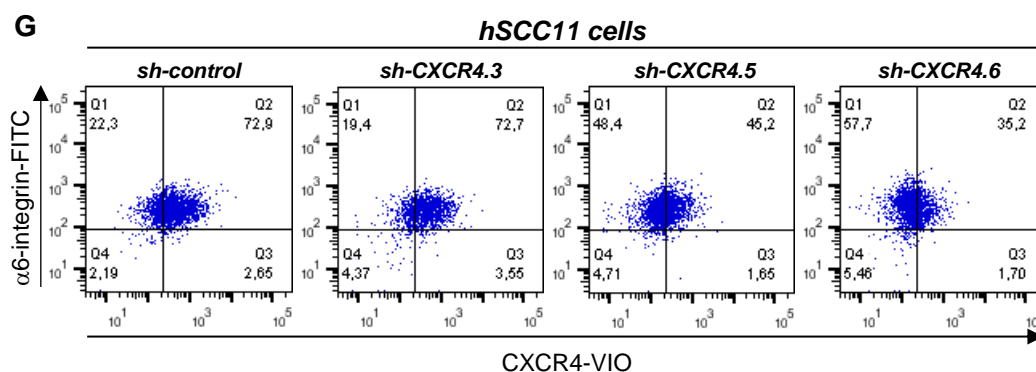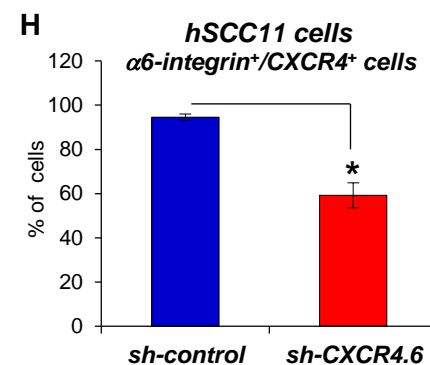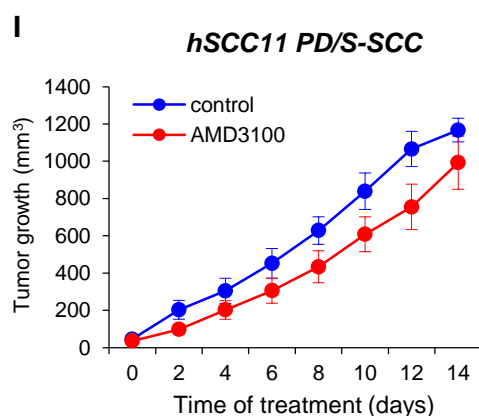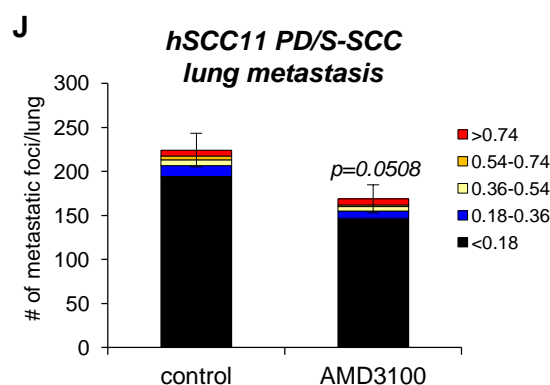

Supplement: Supplementary file 2 — Supplementary Figures. [file 41388_2019_773_MOESM2_ESM.pdf]
